# Mice doubly deficient in *Six4* and *Six5* show ventral body wall defects reproducing human omphalocele

**DOI:** 10.1242/dmm.034611

**Published:** 2018-10-25

**Authors:** Masanori Takahashi, Masaru Tamura, Shigeru Sato, Kiyoshi Kawakami

**Affiliations:** 1 Division of Biology, Center for Molecular Medicine, Jichi Medical University, 3311-1, Yakushiji, Shimotsuke, Tochigi, 329-0498, Japan; 2 Technology and Development Team for Mouse Phenotype Analysis, RIKEN BioResource Center, 3-1-1, Koyadai, Tsukuba, Ibaraki, 305-0074, Japan

**Keywords:** *Six4*/*Six5*, Omphalocele, Primary body wall, Coelomic epithelial cells, Mesothelium, Mouse

## Abstract

Omphalocele is a human congenital anomaly in ventral body wall closure and may be caused by impaired formation of the primary abdominal wall (PAW) and/or defects in abdominal muscle development. Here, we report that mice doubly deficient in homeobox genes *Six4* and *Six5* showed the same ventral body wall closure defects as those seen in human omphalocele. SIX4 and SIX5 were localized in surface ectodermal cells and somatic mesoderm-derived mesenchymal and coelomic epithelial cells (CECs) in the PAW. *Six4^−/−^;Six5^−/−^* fetuses exhibited a large omphalocele with protrusion of both the liver and intestine, or a small omphalocele with protrusion of the intestine, with complete penetrance. The umbilical ring of *Six4^−/−^;Six5^−/−^* embryos was shifted anteriorly and its lateral size was larger than that of normal embryos at the E11.5 stage, before the onset of myoblast migration into the PAW. The proliferation rates of surface ectodermal cells in the left and right PAW and somatic mesoderm-derived cells in the right PAW were lower in *Six4^−/−^;Six5^−/−^* embryos than those of wild-type embryos at E10.5. The transition from CECs of the PAW to rounded mesothelial progenitor cells was impaired and the inner coelomic surface of the PAW was relatively smooth in *Six4^−/−^;Six5^−/−^* embryos at E11.25. Furthermore, *Six4* overexpression in CECs of the PAW promoted ingression of CECs. Taken together, our results suggest that *Six4* and *Six5* are required for growth and morphological change of the PAW, and the impairment of these processes is linked to the abnormal positioning and expansion of the umbilical ring, which results in omphalocele.

## Abstract (precis)

**Summary:** The homeobox genes *Six4* and *Six5* are involved in the regulation of cell proliferation and mesothelium formation in the primary body wall, and *Six4^−/−^;Six5^−/−^* mice are a suitable animal model for human middle-type omphalocele.

## INTRODUCTION

Ventral body wall closure is a crucial process to ensure the storage of internal abdominal organs in the body cavity and to establish the umbilical ring at the central point of the ventral body. In humans, defects in ventral body wall closure cause congenital diseases, including omphalocele, gastroschisis and thoracoabdominoschisis (Brewer and Williams, 2004b; Feldkamp et al., 2007; Williams, 2008). Omphalocele occurs in approximately 1/2000 births (Brewer and Williams, 2004b; Torres et al., 2015) and patients show protrusion of their liver and/or intestine into the umbilical cord with loss of the anterior or posterior abdominal wall. Most infants with a large omphalocele, which is characterized by protrusion of the liver and intestine, have chromosomal defects. Omphalocele is classified into three types: upper-type, middle-type and lower-type, according to the position of the hernia (Duhamel, 1963). Patients with upper-type omphalocele show loss of the anterior body wall and sternum and have cardiac anomaly and diaphragm hernia, whereas patients with lower-type omphalocele have defects in the anus, bladder and external genitalia (Duhamel, 1963; Klein et al., 1981; Stoll et al., 2008). In contrast, middle-type omphalocele, which is predominant in patients with a large omphalocele, usually exhibits no other complex anomalies in other organs, with the exception of loss of the anterior abdominal wall (Duhamel, 1963; Klein et al., 1981; Torres et al., 2015). Body wall closure defects with the protrusion of the intestine into the sac is called ‘hernia into the umbilical cord’ in humans, whereas the phenotype that is similar in mice mutants is categorized as ‘moderate omphalocele’ (Shimizu et al., 2005). We used the term of ‘small omphalocele’ to define omphaloceles with protrusion of the intestine but not the liver, instead of ‘moderate omphalocele’. Omphalocele is thought to be a sporadic disease. The upregulation of *IGF2* by abnormal imprinting causes Beckwith-Wiedemann syndrome, which is characterized by a large body, large internal organs and severe omphalocele (Caspary et al., 1999). In addition, epidemiological studies suggest that certain environmental risk factors increase the occurrence of omphalocele (Mac Bird et al., 2009). However, the genetic factors associated with each type of large omphalocele and the cellular mechanisms of omphalocele formation are still largely unknown.

Two major hypotheses to explain the cause of large omphalocele have been proposed: the primary abdominal wall (PAW) defect theory and the secondary abdominal wall defect theory (see reviews by Brewer and Williams, 2004b; Williams, 2008; Sadler and Feldkamp et al., 2008; Feldkamp et al., 2007; Sadler, 2010; Nichol et al., 2012). The ventral body wall is initially derived from the PAW, which is composed of the somatic mesoderm and surface ectoderm (Durland et al., 2008). Myoblasts that originate from somites migrate into the PAW and differentiate into abdominal muscles to sequentially form the secondary body wall (reviewed in Nichol et al., 2012). A recent study demonstrated the abnormal formation of abdominal muscle in human fetal specimens with a large omphalocele, supporting the secondary abdominal wall defect theory (Nichol et al., 2012). However, notably, most of the patients with a large omphalocele, who are missing the anterior abdominal wall, have no anomalies in abdominal muscle differentiation (Klein et al., 1981). Therefore, other unknown defects in morphogenesis of the PAW may be involved in the occurrence of large omphaloceles in humans.

Various mouse models that show ventral body wall closure defects have been reported (see also reviews by Brewer and Williams, 2004b; Williams, 2008). *Tfap2α* (previously known as *AP-2α*) and *Pitx2* mutants show severe ventral body wall closure defects such as thoracoabdominoschisis and omphalocele (Zhang et al., 1996; Brewer and Williams, 2004a; Kitamura et al., 1999; Gage et al., 1999; Eng et al., 2012). *Msx1/Msx2* double-knockout mice exhibit severe omphalocele, with non-elongation of the PAW and retardation of muscle cell migration (Ogi et al., 2005). These observations suggest that a large omphalocele may be caused by early defects before formation of the secondary body wall. *Fgfr1/Fgfr2* double-knockout mice show small middle-type omphalocele with secondary body wall defects, including disruptions in skin, muscles and connective tissues; however, defects in the PAW have not been examined in detail (Nichol et al., 2011). *Extra-toes* mice, which are spontaneous mutant mice of *Gli3*, a negative regulator of Shh signaling, show polydactyly and a large lower-type omphalocele (Matsumaru et al., 2011). Gain of function for Shh signaling also causes the large lower-type omphalocele phenotype with defects in abdominal muscle development (Matsumaru et al., 2011). These mutant mice show defects in the pelvic girdle and limb development.

In addition to the mutant mice described above, previous studies have revealed that several mouse strains that exhibit the middle-type omphalocele have mutations that disrupt the regulation of cell behaviors such as cell adhesion, cell migration, cell contraction, epithelial-to-mesenchymal transition (EMT) and cell repulsion (Doyonnas et al., 2001; Mesaeli et al., 1999; Rauch et al., 2000; Lian et al., 2012; Shimizu et al., 2005; Thumkeo et al., 2005; Dravis and Henkemeyer, 2011). However, detailed analyses of the developmental processes that underlie omphalocele have not yet been undertaken in these mutant mice. Novel genetic mouse models that resemble the phenotypes of omphalocele patients with no additional defects in organs, only those of the ventral body wall, will offer a great advantage for uncovering the mechanisms of omphalocele formation at the cellular and molecular levels.

In this study, we focus on *Six4/AREC3* and *Six5*, which are categorized as *Six4/Six5* subfamily members (Kawakami et al., 1996a,b) of Six family homeobox transcription factors (SIX1-SIX6) (reviewed by Kawakami et al., 2000; Kumar, 2009). We found that *Six4/Six5* double-homozygous deficient (*Six4^−/−^;Six5^−/−^*) mice exhibited phenotypes that resembled human middle-type omphalocele, including both small and large omphaloceles. We analyzed the morphological defects, cell proliferation and apoptosis in the PAW, and properties of coelomic epithelial cells (CECs) in *Six4^−/−^;Six5^−/−^* embryos. From the results, we propose that the regulation of cell proliferation and morphological change in the PAW at an early stage is a basis for omphalocele phenotype, and that *Six4^−/−^;Six5^−/−^* mice are a suitable animal model for reproducing human middle-type omphalocele.

## RESULTS

### *Six4*/*Six5* double deficiency causes omphalocele in mice

**Fig. 1.**
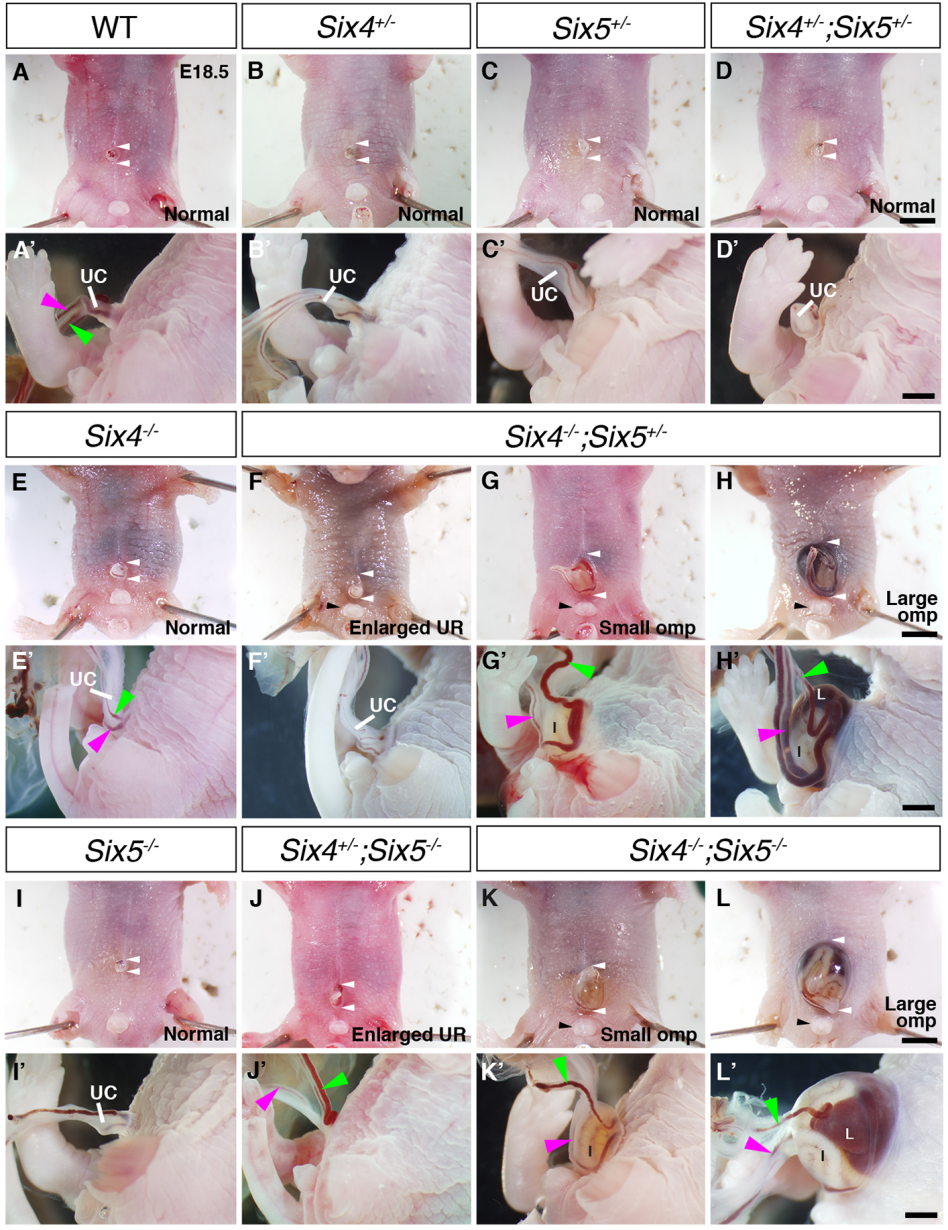
**Abdominal wall closure** **defects in *Six4^−/−^;Six5^+/−^* and *Six4^−/−^;Six5^−/−^* fetuses.** (A-L′) Ventral (A-L) and left side (A′-L′) views of the umbilical region in wild-type (A,A′), *Six4^+/−^* (B,B′), *Six5^+/−^* (C,C′), *Six4^+/−^;Six5^+/−^* (D,D′), *Six4^−/−^* (E,E′), *Six4^−/−^;Six5^+/−^* (F-H′), *Six5^−/−^* (I,I′), *Six4^+/−^;Six5^−/−^* (J,J′) and *Six4^−/−^;Six5^−/−^* fetuses (K-L′) at E18.5. White arrowheads indicate the positions of the anterior and posterior margin of the umbilical ring (UR). Most wild-type and *Six4^−/−^* fetuses exhibit normal fusion of the vitelline (green arrowheads) and umbilical vessels (magenta arrowheads) in the umbilical cord (UC) (A,A′,E,E′). *Six4^−/−^;Six5^+/−^* fetuses show anteriorly enlarged UR (F-H), and fetuses with small or large omphaloceles (omp) have separated vitelline and umbilical vessels (G′,H′). All *Six5^−/−^* fetuses have normal UC and UR (I,I′). A small number of *Six4^+/−^;Six5^−/−^* fetuses have separated vitelline and umbilical vessels (J′), as well as enlarged UR (J). All *Six4^−/−^;Six5^−/−^* fetuses exhibit omphalocele with protrusion of the liver and/or intestine (K-L′). Their vitelline and umbilical vessel do not fuse (K′,L′). The outer genital organ forms normally in *Six4^−/−^;Six5^+/−^* and *Six4^−/−^;Six5^−/−^* fetuses (black arrowhead in F-H,K,L). I, intestine; L, liver. Scale bars: 2 mm in D,H,L; 1 mm in D′,H′,L′.

*Six4^−/−^*, *Six5^−/−^* and *Six4^+/−^;Six5^−/−^* mice are viable, and developmental abnormalities in these mice have not been reported (Klesert et al., 2000; Sarkar et al., 2000; Ozaki et al., 2001; Yajima et al., 2010; Yajima and Kawakami, 2016). SIX4 has a protein structure similar to that of SIX5 (Kawakami et al., 1996a,b), suggesting functional compensation between *Six4* and *Six5*. In fact, overexpression and knockdown of *Six4* and *Six5* in isolated muscle satellite cells demonstrated the common and independent function of *Six4* and *Six5* (Yajima et al., 2010). However, roles of *Six4* and *Six5* during development remain unclear. Therefore, we revisited the phenotypes of fetuses carrying various gene dosages of *Six4* and *Six5* during development. We found that fetuses with all predictable genotypes developed at embryonic day (E)18.5 in the expected Mendelian ratio (Table S1). As we found various types of ventral body wall closure defects in fetuses analyzed at E18.5, we classified them into three types: large omphalocele with the protrusion of the liver and intestine, small omphalocele with the protrusion of the intestine, and enlarged umbilical ring with anteriorly elongated shape of the umbilical ring compared with normal fetuses (Fig. 1, Table S2). A small number of *Six4^−/−^* fetuses exhibited an enlarged umbilical ring at E18.5 (Table S2), but others showed no abnormalities in the ventral body wall (Fig. 1E,E′). Although all *Six5^−/−^* fetuses had no phenotypic changes in the ventral body (Fig. 1I,I′), a small number of *Six4^+/−^;Six5^−/−^* fetuses showed an enlarged umbilical ring or a small-type omphalocele (Fig. 1J,J′). Remarkably, most of the *Six4^−/−^;Six5^+/−^* fetuses had ventral body closure defects, including the omphalocele phenotype, at E18.5 (Fig. 1F-H′) (enlarged umbilical ring, *n*=9/16, 56.3%; small omphalocele, *n*=3/16, 18.8%; large omphalocele, *n*=2/16, 12.5%; Table S2). All of the *Six4^−/−^;Six5^−/−^* fetuses exhibited small or large omphaloceles at E18.5 (Fig. 1K-L′) (small omphalocele, *n*=9/28, 32.1%; large omphalocele, *n*=19/28, 67.9%; Table S2) with complete penetrance. These results indicate that *Six4* and *Six5* together are indispensable for ventral body wall closure in mice and that *Six4* is the primary player between the two.

Patients with omphalocele with severe protrusion of the liver and intestine often show other malformations at the thoracic and lumbar levels (Stoll et al., 2008; Torres et al., 2015). Therefore, we next examined whether the fetuses described above have the defects in their skeletal structure, such as the skull, sternums and vertebrae (Fig. S1). *Six5^−/−^* and *Six4^+/−^;Six5^−/−^* fetuses had normal skeletal structure (Fig. S1A-B′). In *Six4^−/−^;Six5^−/−^* fetuses, the numbers of ribs and sternums were normal, but formation of the ensiform cartilage was impaired (Fig. S1C-D′). These results indicate that the defects in *Six4^−/−^;Six5^−/−^* fetuses are mostly confined to the abdominal body wall.

### Abdominal muscles and their progenitors present normally in the abdominal wall of *Six4/Six5* double-deficient mice

Previous studies have shown that abnormal formation of the secondary abdominal wall, including abdominal muscle differentiation, may be the cause of the large omphalocele phenotype in humans (Nichol et al., 2012) and in mouse models (Ogi et al., 2005; Nichol et al., 2011). Therefore, we examined defects of the abdominal muscle differentiation in *Six4^−/−^;Six5^−/−^* mice during abdominal wall formation.

We compared ventral body wall formation in *Six4^−/−^;Six5^−/−^* fetuses with large omphalocele and *Six5^−/−^* or *Six4^+/−^;Six5^+/−^* fetuses, which have no apparent defects in ventral body wall closure, at E15.5 and E18.5 (Figs S2 and S3). The abdominal wall was closed at the umbilical cord level and it completely covered internal organs at the level anterior to the umbilical cord in *Six5^−/−^* fetuses (Fig. S2A,I). The herniated livers and intestines in *Six4^−/−^;Six5^−/−^* fetuses were covered with the peritoneum and amnion at the umbilical cord level (Fig. S2J). The anterior abdominal wall was completely lost in *Six4^−/−^;Six5^−/−^* fetuses (Fig. S2B).

Myoblasts start to migrate into the PAW at E10.5 and differentiate into four types of hypaxial muscles, specifically the transversus abdominis muscle (TA), internal oblique muscle (IO), external oblique muscle (EO) and rectus abdominis muscle (RA), by E13.5 (Nichol et al., 2012). These muscles are identified by the expression of myosin-heavy chain-II (MyHC-II; also known as Myh2). TA, IO, EO and RA were positioned in *Six4^−/−^;Six5^−/−^* fetuses in a pattern similar to that observed in *Six5^−/−^* fetuses at E18.5 (Fig. S2E-H,M-P). The epaxial extensor muscle (Fig. S2C,D,K,L) and panniculus carnosus muscle (PC) were normally positioned in *Six4^−/−^;Six5^−/−^* fetuses and *Six5^−/−^* fetuses (Fig. S2E-H,M-P). All abdominal muscles were separated and positioned in the ventral wall of *Six4^−/−^;Six5^−/−^* embryos in a pattern similar to that in control *Six4^+/−^;Six5^+/−^* embryos at E15.5 (Fig. S3A-H). Previous studies have shown that SIX family proteins are involved in skeletal muscle differentiation by activating the expression of muscle regulatory factors MYOG and MYOD1 (Spitz et al., 1998; Relaix et al., 2013). MYOG was expressed in abdominal muscle cells in *Six4^+/−^;Six5^+/−^* and in *Six4^−/−^;Six5^−/−^* embryos with similar patterns at E15.5 (Fig. S3I-N). In addition, MYOG and MYOD1 were expressed in abdominal muscle cells in *Six4^+/−^;Six5^+/−^* and *Six4^−/−^;Six5^−/−^* embryos with similar patterns, except the downregulation in the PC at E13.5 (Fig. S4). These results suggest that there are no severe defects in abdominal muscle differentiation in *Six4^−/−^;Six5^−/−^* embryos. Our results indicate that omphalocele formation in *Six4^−/−^;Six5^−/−^* mice is not attributable to impaired abdominal muscle differentiation and clearly suggest the other causes of large omphalocele.

### Positioning of the umbilical ring and anterior PAW formation are impaired in *Six4/Six5* double-deficient mice

**Fig. 2.**
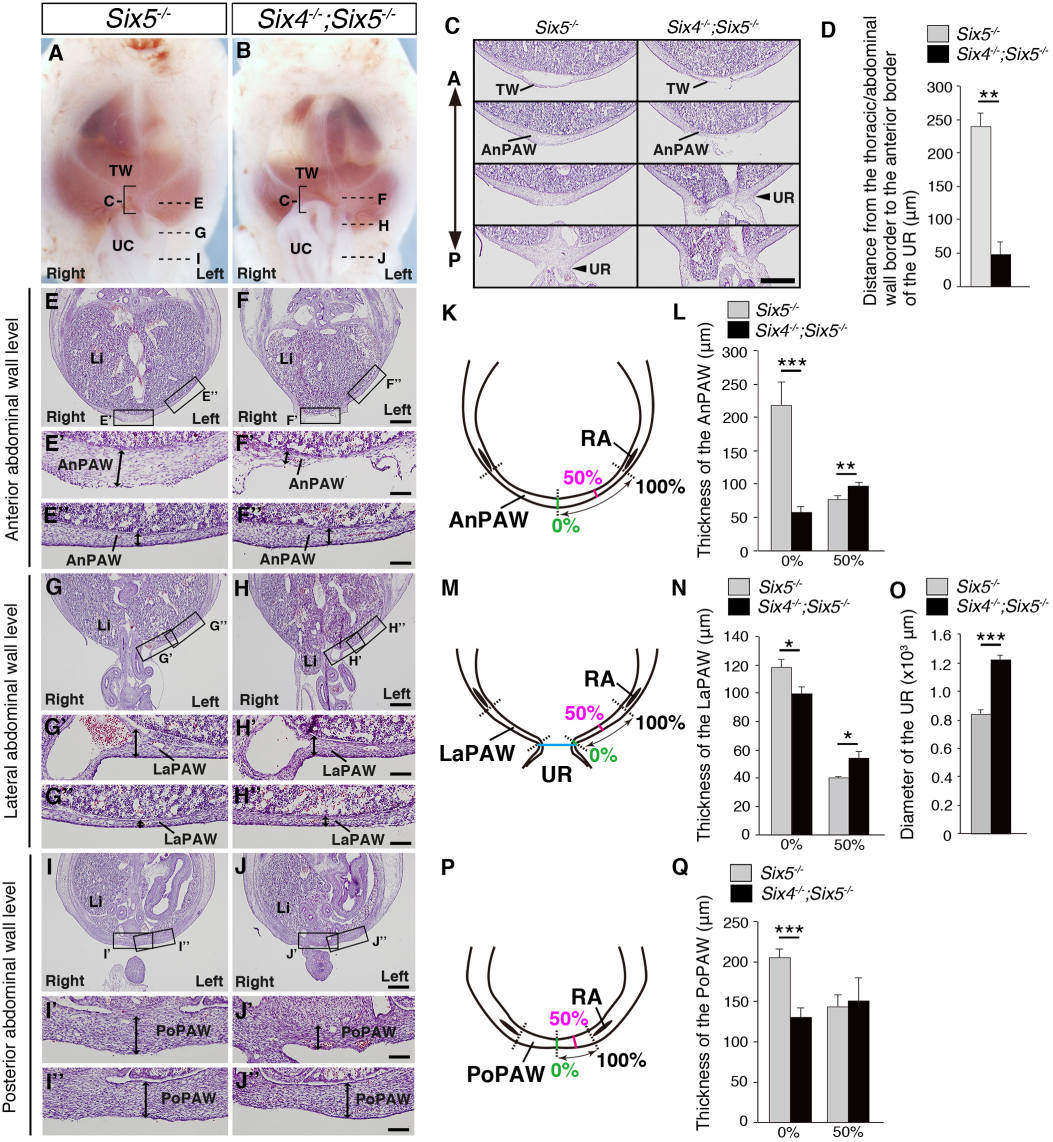
**Enlarged umbilical ring and** **impaired formation of the anterior abdominal wall in *Six4^−/−^;Six5^−/−^* embryos.** (A-J″) Whole-mount preparation to observe the umbilical region (A,B) and histological analysis at E13.0 (C,E-J″). The head, forelimbs and hindlimbs are removed in each embryo. Panels in C show cross sections of the bracketed area in A and B at four different levels along the anterior-posterior (A↔P) axis of *Six5^−/−^* (left) and *Six4^−/−^;Six5^−/−^* (right) embryos: from the most anterior thoracic wall to the most-posterior anterior abdominal wall. The anterior border of the umbilical ring (UR, black arrowhead) shifts anteriorly in *Six4^−/−^;Six5^−/−^* embryos compared with that of *Six5^−/−^* embryos (C,D). Sections of E,F,G,H,I,J correspond to the levels indicated with E,G,I (in A) and F,H,J (in B). Images in E′,E″,F′,F″,G′,G″,H′,H″,I′,I″,J′,J″ show higher magnifications of the boxed areas in E,F,G,H,I,J. The thickness of the AnPAW is reduced in *Six4^−/−^;Six5^−/−^* embryos at the 0% position (↔ in F′) and increased at the 50% position (↔ in F″) compared with that of *Six5^−/−^* embryos (↔ in E′,E″). The liver (Li) invades the umbilical cord in *Six4^−/−^;Six5^−/−^* embryos (H) but not in *Six5^−/−^* embryos (G). The thickness of the LaPAW in *Six4^−/−^;Six5^−/−^* embryos is smaller than that of *Six5^−/−^* embryos at the 0% position (↔ in G′,H′) and larger than that of *Six5^−/−^* embryos at the 50% position (↔ in G″,H″). The thickness of the PoPAW is reduced in *Six4^−/−^;Six5^−/−^* embryos at the 0% position (↔ in J′) compared with that of *Six5^−/−^* embryos (↔ in I′) and is similar at the 50% position (↔ in I″,J″). (K-Q) Thickness of the AnPAW (L), LaPAW (N) and PoPAW (Q) at the left side of the embryos. Schematics showing positions of the measurement of abdominal wall thickness at the different levels and distance of the UR (K,M,P). O indicates the cross-sectional diameter of the UR. Error bars show standard errors. *P<0.05, **P<0.01, ***P<0.001, Student's *t*-test, *n*=3. RA, rectus abdominis; TW, thoracic wall; UC, umbilical cord. Scale bars: 500 μm in C,E-J; 100 μm in E′-J′,E″-J″.

Because abdominal myoblasts normally differentiate into muscles in *Six4^−/−^;Six5^−/−^* embryos, defects in PAW formation have been considered as the cause of omphalocele. Therefore, we next examined the effects of *Six4* deficiency in a *Six5*-null background on PAW formation along the anterior-posterior axis in comparison with the phenotypes of control (*Six5^−/−^*) and *Six4^−/−^;Six5^−/−^* embryos at E13.0 (Fig. 2). During development in *Six5^−/−^* embryos, the posterior border of the thoracic wall was separated from the anterior border of the umbilical ring by E13.0, and the anterior PAW (AnPAW) was formed in the region posterior to the thoracic diaphragm (*n*=3, Fig. 2A,C). The umbilical ring was positioned anteriorly in E13.0 *Six4^−/−^;Six5^−/−^* embryos (48±19.6 μm from the thoracic/abdominal wall border, mean±s.e.m., *n*=3) (Fig. 2B-D) in contrast to *Six5^−/−^* embryos (240±19.6 μm from the thoracic/abdominal wall border; *P*=0.0048, Student's *t*-test, *n*=3) (Fig. 2A,C,D). The AnPAW of *Six4^−/−^;Six5^−/−^* embryos was markedly thinner than that of *Six5^−/−^* embryos at the 0% position of the AnPAW (*Six5^−/−^*, 217.7±35.2 μm; *Six4^−/−^;Six5^−/−^*, 57.6±9.3 μm; *P*=0.00064, Student's *t*-test, *n*=3) but thicker than that of *Six5^−/−^* embryos at the 50% position of the AnPAW (*Six5^−/−^*, 76.7±5.3 μm; *Six4^−/−^;Six5^−/−^*, 96.9±5.9 μm; *P*=0.0064, Student's *t*-test, *n*=3) (Fig. 2E-F″,K,L). The lateral PAW (LaPAW) of *Six4^−/−^;Six5^−/−^* embryos was thinner than that of *Six5^−/−^* embryos at the 0% position of the LaPAW (*Six5^−/−^*, 118.2±6.3 μm; *Six4^−/−^;Six5^−/−^*, 99.6±5.1 μm; *P*=0.027, Student's *t*-test, *n*=3) but thicker than that of *Six5^−/−^* embryos at the 50% position of the LaPAW (*Six5^−/−^*, 40.2±1.2 μm; *Six4^−/−^;Six5^−/−^*, 54.1±4.7 μm; *P*=0.0106, Student's *t*-test, *n*=3) (Fig. 2G-H″,M,N). The liver was partially protruded into the umbilical area at the right side in *Six4^−/−^;Six5^−/−^* embryos (*n*=3/3, Fig. 2G,H). The cross-sectional diameter of the umbilical ring of *Six4^−/−^;Six5^−/−^* embryos was larger than that of *Six5^−/−^* embryos (*Six5^−/−^*, 840.2±26.3 μm; *Six4^−/−^;Six5^−/−^*, 1217.4.7±39.8 μm; *P*=1.13E-08, Student's *t*-test, *n*=3) (Fig. 2G,H,M,O). The thickness of the posterior PAW (PoPAW) of *Six4^−/−^;Six5^−/−^* embryos was significantly smaller at the 0% position (*Six5^−/−^*, 205.1±10.8 μm; *Six4^−/−^;Six5^−/^*, 130.2±11.0 μm; *P*=0.00047, Student's *t*-test, *n*=3) but similar at the 50% position of the PoPAW (*Six5^−/−^*, 143.8±15.7 μm; *Six4^−/−^;Six5^−/−^*, 151.2±29.0 μm; *P*=0.810, Student's *t*-test, *n*=3) to that in *Six5^−/−^* embryos (Fig. 2I-J″,P,Q). These results suggest that *Six4/Six5* are required for correct positioning of the umbilical ring and PAW morphogenesis in the anterior, lateral and posterior abdominal regions.

### Micro-CT scanning analyses reveal early morphological changes in the PAW of *Six4/Six5* double-deficient mice

**Fig. 3.**
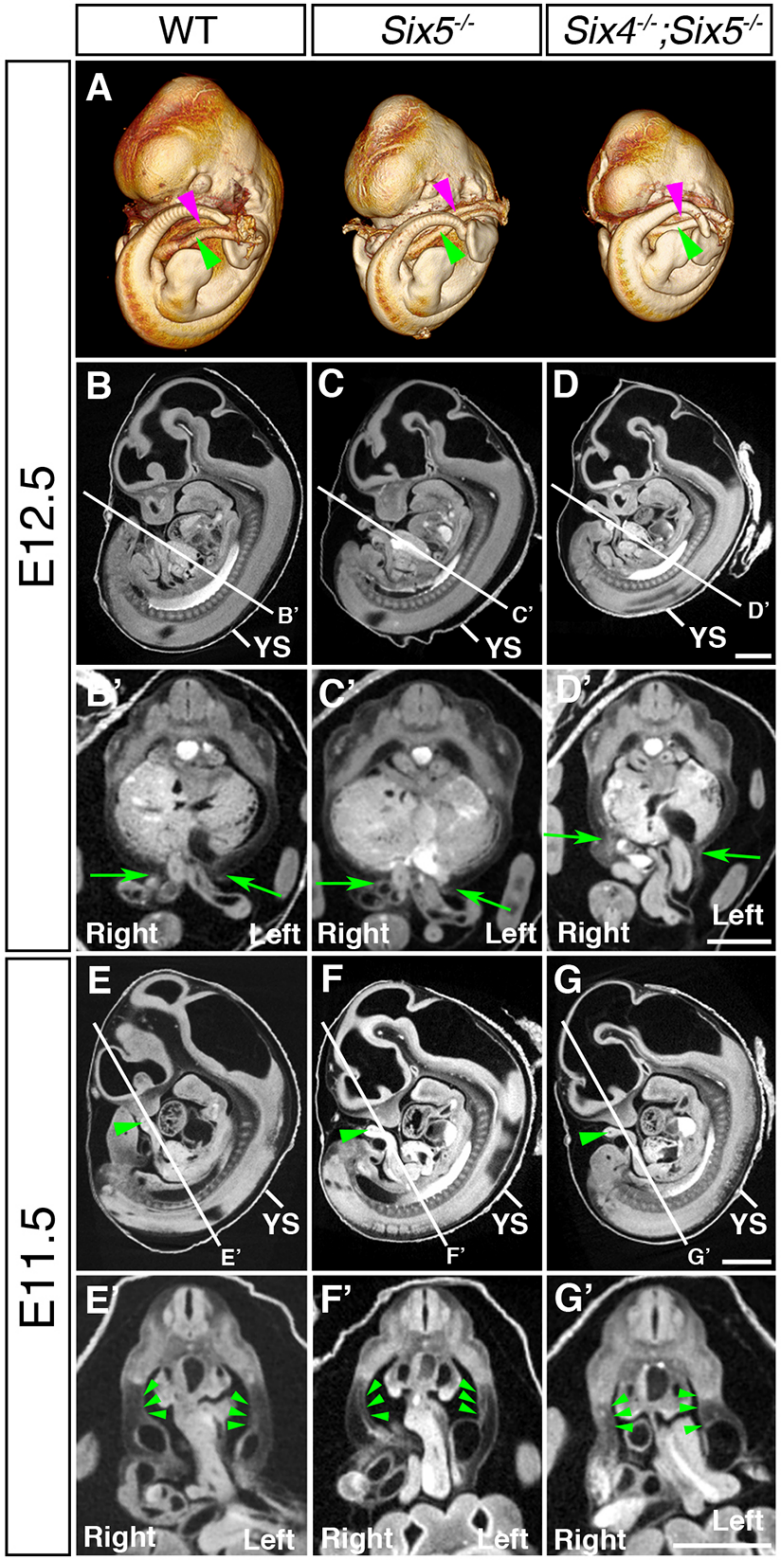
**Micro-CT analysis of wild-type, *Six5^−/−^* and *Six4^−/−^;Six5^−/−^* embryos.** Embryos with the placenta and amnion were fixed for the analysis. (A) Three-dimensional analysis at E12.5 reveals that *Six4^−/−^;Six5^−/−^* embryos exhibit normal umbilical cord formation. Umbilical veins (magenta arrowheads) and arteries (green arrowheads) ­are indicated. (B-D′) The umbilical ring of *Six4^−/−^;Six5^−/−^* embryos is enlarged compared with that of *Six5^−/−^* and wild-type embryos (between green arrows in B′,C′,D′) at E12.5. (E-G′) Looping of the midgut is normal in *Six4^−/−^;Six5^−/−^* embryos (green arrowheads in E-G) at E11.5. No bending of the PAW at the distal levels is detected in *Six4^−/−^;Six5^−/−^* embryos, in contrast to wild-type and *Six5^−/−^* embryos (green arrowheads in E′,F′,G′) at E11.5. YS, yolk sac. Scale bars: 1 mm in B-G′.

To analyze the detailed three-dimensional structure of the umbilical region, we performed micro-CT scanning analysis, which is a powerful method to identify structural phenotypes in mutant mice even at the early stages (Dickinson et al., 2016) (Fig. 3). To retain the structure of the umbilical region, we isolated embryos with yolk sacs, placentas and intact umbilical vessels from the uteruses of pregnant mice for micro-CT analyses. Three-dimensional analysis of embryos revealed that the umbilical cords, which contained an artery and a vein, developed and were positioned normally in *Six4^−/−^;Six5^−/−^* embryos (*n*=3) with patterns similar to wild-type and *Six5^−/−^* embryos at E12.5 (*n*=3 and 2, respectively, Fig. 3A). Notably, enlargement of the umbilical ring was observed in *Six4^−/−^;Six5^−/−^* embryos (*n*=3, Fig. 3D′) compared with wild-type and *Six5^−/−^* embryos at E12.5 (*n*=3 and 3, respectively, Fig. 3B′,C′). However, the liver did not protrude from the abdominal cavity in all *Six4^−/−^;Six5^−/−^* embryos (*n*=3, Fig. 3D′; Movies 1 and 2). These results suggest that umbilical ring formation is impaired in *Six4^−/−^;Six5^−/−^* embryos before the protrusion of the liver.

We next examined the apparent structural defects in the umbilical regions in *Six4^−/−^;Six5^−/−^* embryos at the stage before E12.5. Physiological herniation and looping of the midgut were observed in wild-type and *Six5^−/−^* embryos (*n*=3 and 4, respectively, Fig. 3E,F; Movie 3), and *Six4^−/−^;Six5^−/−^* embryos showed similar patterns to wild-type and *Six5^−/−^* embryos at E11.5 (*n*=2, Fig. 3G; Movie 4). Remarkably, bending of the PAW was missing at the distal levels in *Six4^−/−^;Six5^−/−^* embryos at E11.5 (*n*=1/2, Fig. 3G′) in contrast to wild-type and *Six5^−/−^* embryos (Fig. 3E′,F′). Taken together, our results suggest that the ventral closure defects in *Six4^−/−^;Six5^−/−^* embryos are not associated with initial formation of the midgut, and that loss of *Six4/Six5* affects the morphogenesis of the PAW and size of umbilical ring at E11.5.

### SIX4 and SIX5 are localized in PAW cells, but SIX4 expression is transient

**Fig. 4.**
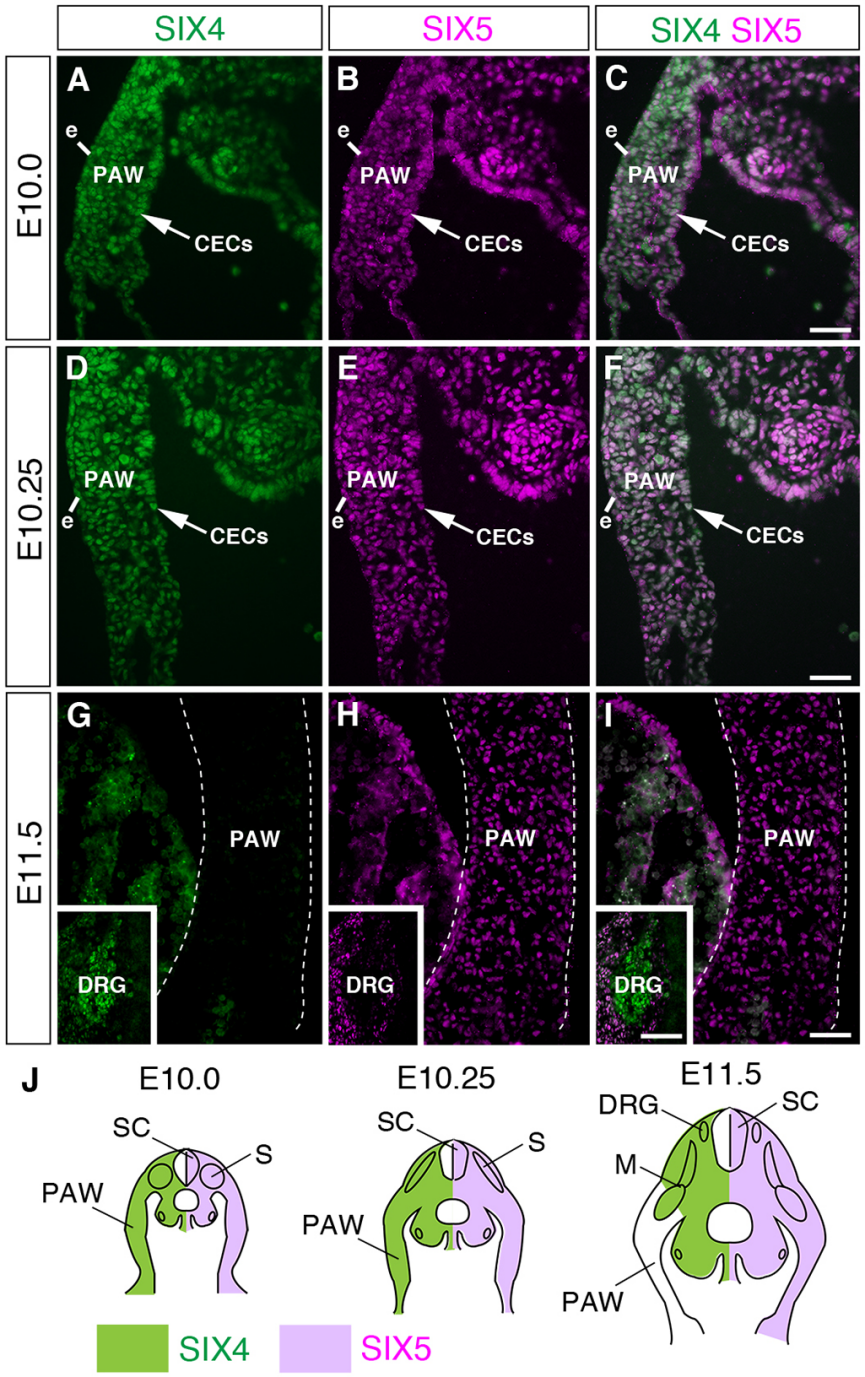
**Localization of the SIX4 and SIX5 proteins in the PAW.** (A-F) SIX4 and SIX5 proteins localize in surface ectodermal cells, CECs (arrow) and mesenchymal cells in the PAW at E10.0 (A-C) and E10.25 (D-F). (G-I) SIX5 but not SIX4 is detected in the PAW at E11.5. Insets in G-I indicate the localization of SIX4 and SIX5 in the dorsal root ganglia. Dotted lines indicate inner and outer surfaces of the PAW. (J) Illustration of localization patterns of SIX4 and SIX5 at E10.0, E10.25 and E11.5. DRG, dorsal root ganglia; e, ectoderm; M, muscle progenitors; S, somite; SC, spinal cord. Scale bars: 50 μm in A-I; 25 μm in inset of G-I.

The expression of *Six4* in the early PAW has been reported (Kobayashi et al., 2007), but the expression of *Six5* during development has not been analyzed in detail. Furthermore, the localization patterns of SIX4 and SIX5 proteins have not been characterized. Therefore, we next examined the localization of the SIX4 and SIX5 proteins in the PAW using specific antibodies (Yajima et al., 2010) at E10.0-E11.5 (Fig. 4). SIX4 and SIX5 localized in surface ectodermal cells, mesenchymal cells and CECs, which overlay the mesenchyme and differentiate into mesothelial cells, at E10.0-E10.25 (Fig. 4A-F, Fig. S5C,L). The overlay images of SIX4 and SIX5 immunofluorescence indicated that individual PAW cells showed the differential expression levels of SIX4 and SIX5 (Fig. 4C,F). In contrast to SIX5 localization at E11.5, SIX4 was not observed in the PAW at E11.5 (Fig. 4G-I). Expression of SIX4 and SIX5 in the PAW was detected at earlier stages (E9.25-E9.5) (Fig. S5A,B,J,K). As SIX1, another member of the Six family protein, is known to compensate for the roles of SIX4 in other tissues, we examined possible contribution of SIX1 in PAW development. However, SIX1 was not detected in the PAW at E10.75 (Fig. S6A). Consistent with protein localization, *Six4* mRNA was completely absent in the PAW (Fig. S7B,E) and *Six5* mRNA was expressed in the PAW at E11.5 and E12.5 (Fig. S7C,F). These results suggest that the expressions of *Six4* and *Six5* are differentially regulated in the PAW and they cooperatively function in the PAW at a stage before E11.5.

### Cell proliferation in the PAW is altered in *Six4/Six5* double-deficient mice

**Fig. 5.**
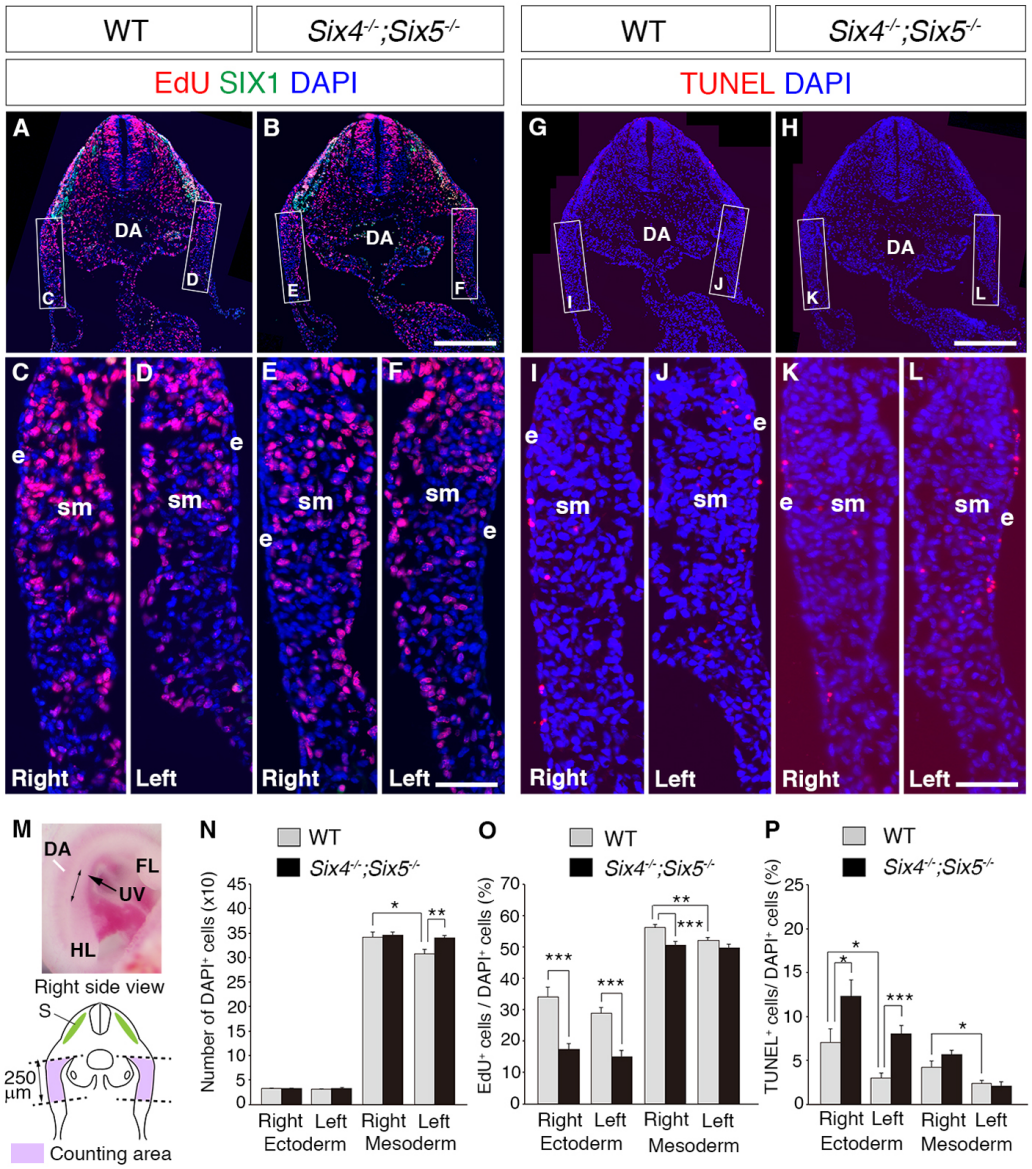
**Altered cell proliferation rate in the PAW of *Six4^−/−^;Six5^−/−^* embryos.** (A-F) Distribution of EdU-positive cells after EdU incorporation for 30 min in the PAW of wild-type (A,C,D) and *Six4^−/−^;Six5^−/−^* (B,E,F) embryos at E10.5. The SIX1 signal, which is used as a marker for muscle progenitor cells, is observed in wild-type and *Six4^−/−^;Six5^−/−^* embryos (A,B). (G-L) Distribution of TUNEL-positive apoptotic cells in the PAW of wild-type (G,I,J) and *Six4^−/−^;Six5^−/−^* (H,K,L) embryos at E10.5. (M) A right-side view of an E10.5 mouse embryo. A double-headed arrow (↔) indicates the anterior-posterior level of the analyzed sections. The illustration shows the counting area of EdU- and TUNEL-positive cells in the PAW (pink). (N) The number of cells within the counting area. The number of mesodermal cells in the left side is smaller than that in the right side of the PAW. (O) The ratio of proliferating cells in the PAW. The numbers of EdU-positive cells in the ectodermal and mesodermal cells of the right and left PAW, and the ratio and total cell number were calculated. In wild-type embryos, the ratio of proliferating cells in mesodermal regions of the PAW is higher in the right side than in the left side. In *Six4^−/−^;Six5^−/−^* embryos, the ratio of proliferating cells in the ectodermal region of the PAW is lower in both right and left sides of the PAW than that in the wild-type embryos, whereas the ratio of mesodermal cells is lower in the right side but similar in the left side compared with wild-type embryos. (P) The ratio of TUNEL-positive cells. In wild-type embryos, the ratio of TUNEL-positive cells in the ectodermal and mesodermal regions of the PAW are larger in the right side than that in the left side. The ratio of TUNEL-positive cells in *Six4^−/−^;Six5^−/−^* embryos is larger than that of wild-type embryos in the right and left ectoderm of the PAW. Error bars show the standard error. *P<0.05, **P<0.01, ***P<0.001, Student's *t*-test, *n*=3. DA, dorsal aorta; e, ectoderm; FL, forelimb; HL, hindlimb; S, somite; sm, somatic mesoderm; UV, umbilical vein. Scale bars: 250 μm in A,B,G,H; 50 μm in C-F,I-L.

As formation of the umbilical ring was impaired before the migration of muscle progenitor cells into the PAW, we next hypothesized that impaired cell proliferation in the PAW may cause abnormal PAW morphogenesis. To examine the cell proliferation rate in the PAW at the anterior to middle levels, we labeled proliferating cells with 5-ethynyl-2′-deoxyuridine (EdU) at E10.5 and counted the numbers of EdU-positive cells after 30 min within a 250 μm area from the lateral somitic frontier of the PAW (Fig. 5M).

The total number of surface ectodermal cells was similar between the right and left sides of the wild-type PAW (right, 32.7±0.8 cells; left, 31.1±1.0 cells; *P*=0.22, Student's *t*-test, *n*=3) (Fig. 5C,D,M,N), and there were no significant differences between wild-type and *Six4^−/−^;Six5^−/−^* embryos (right, 32.8±1.2 cells, *P*=0.91; left, 33.1±2.0 cells, *P*=0.15; Student's *t*-test, *n*=3) (Fig. 5C-F,M,N). In contrast, the total number of cells in the somatic mesoderm of the wild-type PAW was higher in the right side than in the left side (right, 342.2±10.6 cells; left, 307.4±8.8 cells; *P*=0.019, Student's *t*-test, *n*=3) (Fig. 5C,D,M,N). The number of mesodermal cells in the right side of *Six4^−/−^;Six5^−/−^* embryos was similar to that in the right side of wild-type embryos (right, 345.8±6.1 cells; *P*=0.80, Student's *t*-test, *n*=3) (Fig. 5C,E,M,N). The number of mesodermal cells in the left side of *Six4^−/−^;Six5^−/−^* embryos was higher than in wild-type embryos (left, 340±4.5 cells; *P*=0.0039, Student's *t*-test, *n*=3) (Fig. 5D,F,M,N), resulting in the disappearance of the left-right asymmetrical ratio.

In the ectoderm of the PAW of wild-type embryos, approximately 30% of cells were EdU-positive, and there were no significant differences between the right and left side of the PAW (right, 34.1±3.1%; left, 29.0±1.8%; *P*=0.17, Student's *t*-test, *n*=3) (Fig. 5A,C,D,O). The ratio of EdU-positive cells to DAPI-positive cells in the ectoderm of the PAW was significantly lower on the right and left sides of *Six4^−/−^;Six5^−/−^* embryos (right, 17.3±1.9%, *P*=0.0001; left, 15.0±2.1%, *P*=0.000023; Student's *t*-test, *n*=3) than those in wild-type embryos (Fig. 5B,E,F,O). The ratio of EdU-positive cells to DAPI-positive cells in the right somatic mesoderm-derived region of the PAW including the mesenchymal cells and CECs was significantly higher than that in the left side of the PAW of wild-type embryos (right, 56.2±0.95%; left, 52.2±0.92%; *P*=0.0057, Student's *t*-test, *n*=3) (Fig. 5A,C,D,O). In *Six4^−/−^;Six5^−/−^* embryos, the ratio of EdU-positive cells to DAPI-positive cells in the somatic mesoderm-derived region of the PAW was lower than that in wild-type embryos in the right side but was similar to wild-type embryos in the left side (right, 50.7±1.1%, *P*=0.00067; left, 49.8±1.1%, *P*=0.11; Student's *t*-test, *n*=3), resulting in the disappearance of the left-right asymmetrical ratio (*P*=0.57, Student's *t*-test, *n*=3) (Fig. 5B,E,F,O). These results suggest that *Six4* and *Six5* are required for the promotion of cell proliferation of ectodermal cells of the right and left PAW and somatic mesodermal-derived cells of the right PAW.

We also examined whether the loss of *Six4* and *Six5* enhances cell death frequency in the PAW (Fig. 5M,P). Most terminal deoxynucleotidyl transferase-mediated dUTP nick end labeling (TUNEL)-positive cells were observed in the surface ectoderm and in the somatic mesoderm-derived region in the PAW of wild-type embryos at E10.5 (Fig. 5G,I,J). In the ectodermal region of the PAW, the ratio of apoptotic cells was higher in the right side than in the left side of the wild-type embryos (right, 7.0±1.5%; left: 3.0±0.6%; *P*=0.03, Student's *t*-test, *n*=3) (Fig. 5G,I,J,P). In the ectodermal region of *Six4^−/−^;Six5^−/−^* embryos, the ratio of TUNEL-positive cells was higher than that in wild-type embryos in the left and right sides (right, 12.3±1.9%, *P*=0.04; left, 8.0±1.0%, *P*=0.00015; Student's *t*-test, *n*=3) (Fig. 5H,K,L,P). In the somatic mesoderm-derived region of the PAW of wild-type embryos, the ratio of apoptotic cells was higher in the right side than in the left side (right, 4.2±0.7%; left, 2.3±0.4%; *P*=0.03, Student's *t*-test, *n*=3) (Fig. 5G,I,J,P). The similar ratio of TUNEL-positive cells in the somatic mesoderm-derived region was observed in each side of the PAW of *Six4^−/−^;Six5^−/−^* embryos (right, 5.6±0.5%, *P*=0.13; left, 2.0±0.5%, *P*=0.6; Student's *t*-test, *n*=3) (Fig. 5H,K,L,P) compared with wild-type embryos. These results suggest that the loss of *Six4* and *Six5* decreased the survival rate of ectodermal cells in the PAW.

### Mesothelium formation is impaired in the PAW of *Six4/Six5* double-deficient mice

**Fig. 6.**
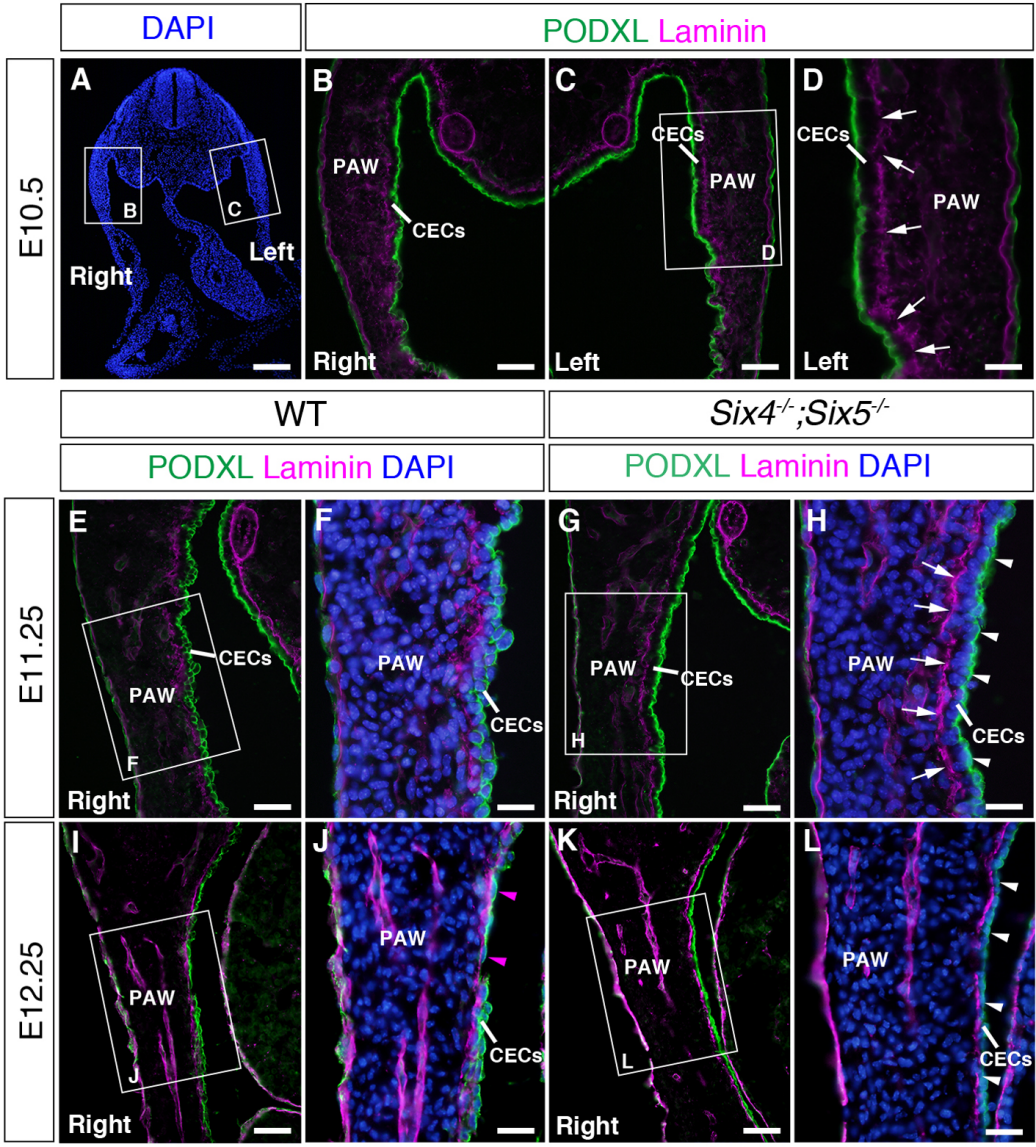
**Transition of CECs to mesothelial progenitor cells in wild-type embryos and impaired mesothelium formation in *Six4^−/−^;Six5^−/−^* embryos.** (A-D) Distribution of laminin and PODXL in wild-type embryos at E10.5. B, C and D are magnified images of the boxed regions in A and C. Laminin localizes at the border between the mesenchymal region and the CE in the right (B) and left side (C, arrows in D) of the PAW. Note that laminin accumulates with a fragmented pattern at the border between the mesenchymal region and the CE in the right (B) and left side (C, arrows in D) at E10.5. PODXL accumulates in the apical side of CECs in the right (B) and left side (C,D). (E-H) Distribution of laminin and PODXL in the PAW of wild-type (E,F) and *Six4^−/−^;Six5^−/−^* embryos at E11.25 (G,H). F and H are magnified images of the boxed regions in E and G, respectively. PODXL distributes at the cell periphery of CECs at E11.25 (E,F). In *Six4^−/−^;Six5^−/−^* embryos, PODXL localizes in the apical domain of CECs in the PAW (arrowheads in H) and laminin accumulates with a less fragmented pattern (arrows in H) in contrast to wild-type embryos (F). (I-L) Distribution of laminin and PODXL in the PAW of wild-type (I,J) and *Six4^−/−^;Six5^−/−^* (K,L) embryos at E12.25. J and L are magnified images of the boxed regions in I and K, respectively. Cells with PODXL at their periphery and cells with squamous shape (arrowheads in J) are observed in the CE of wild-type PAW (J). PODXL localizes in the apical domain of CECs in the PAW of *Six4^−/−^;Six5^−/−^* embryos (arrowheads in L). Scale bars: 200 μm in A; 50 μm in B,C,E,G,I,K; 25 μm in D,F,H,J,L.

Because morphological change in the PAW was observed in *Six4^−/−^;Six5^−/−^* embryos at E11.5 (Fig. 3) as shown above, we reasoned that alterations to the properties of somatic mesoderm-derived tissues had already occurred by E11.5. In coelomic organs, CECs are derived from the somatic or splanchnic mesoderm and finally differentiate into the mesothelial cells and form a monolayer mesothelium at later embryonic and adult stages (Winters and Bader, 2013; Ariza et al., 2016). CECs in the gonad-forming region show growth and EMT to form gonad cells during E9.5-E10.5 in mice (Kusaka et al., 2010; Fujimoto et al., 2013; Tanaka and Nishinakamura, 2014). In contrast to the gonad-forming region, involvement of EMT to form mesenchymal cells is poorly characterized in the abdominal CECs. Therefore, we analyzed properties of CECs in the PAW by examining the distribution of laminin and PODXL (also known as gp135), which is a member of the CD34 family transmembrane sialoglycoproteins (reviewed in Nielsen and McNagny, 2008) and used as a marker for splanchnic mesoderm-derived mesothelial cells (Onitsuka et al., 2010) (Fig. 6).

Localization of laminin, a component of the basement membrane, at the border between the coelomic epithelium (CE) and mesenchyme was observed at E10.25 (Fig. S5H,I) but not at E9.25 and E9.5 (Fig. S5D-G). Laminin was accumulated with a fragmented distribution at the right and left sides of the PAW in wild-type embryos at E10.5 (Fig. 6A-D), and the fragmented distribution was still observed at E11.25 (Fig. 6E,F). PODXL localized in the apical side of CECs in the PAW at E9.25-E10.5 (Figs S5M-R, Fig. 6B-D). Interestingly, CECs were round, with peripheral localization of PODXL, in the PAW of wild-type embryos at E11.25 (Fig. 6E,F). We regarded that the distribution change in PODXL marks the transition from CECs into mesothelial progenitor cells in analogy to the splanchnic mesoderm-derived mesothelial cells (Onitsuka et al., 2010; Winters and Bader, 2013). Remarkably, in *Six4^−/−^;Six5^−/−^* embryos, the distribution of laminin was less fragmented at the border between the CE and mesenchymal cells at E11.25 (*n*=2, Fig. 6G,H). PODXL still localized to the apical side of the membrane of CECs in *Six4^−/−^;Six5^−/−^* embryos at E11.25 (*n*=2, Fig. 6G,H). In addition, the inner coelomic surface of the PAW showed a rugged pattern in wild-type embryos, whereas that of *Six4^−/−^;Six5^−/−^* embryos was relatively smooth (Fig. 6E,G). At E12.25, cells with PODXL at the cell periphery were still observed in the CE of wild-type PAW (*n*=2, Fig. 6I,J). In addition, some CECs showed a squamous shape (Fig. 6I,J). In contrast, PODXL remained localized in the apical domain of CECs in the PAW of *Six4^−/−^;Six5^−/−^* embryos (*n*=2, Fig. 6K,L). These observations suggest that transition of CECs to mesothelial progenitor cells in the PAW was impaired in *Six4^−/−^;Six5^−/−^* embryos. Taken together, our results suggest that the mesothelium formation of the abdominal wall is disturbed before E11.5 in *Six4^−/−^;Six5^−/−^* embryos, and that *Six4* and *Six5* are required for the transition of CECs to round-shaped mesothelial progenitor cells in the PAW and morphogenesis of the inner coelomic surface of the PAW from E10.5 to E11.25.

### *Six4* overexpression in CECs promotes ingression of these cells into the mesenchymal region

**Fig. 7.**
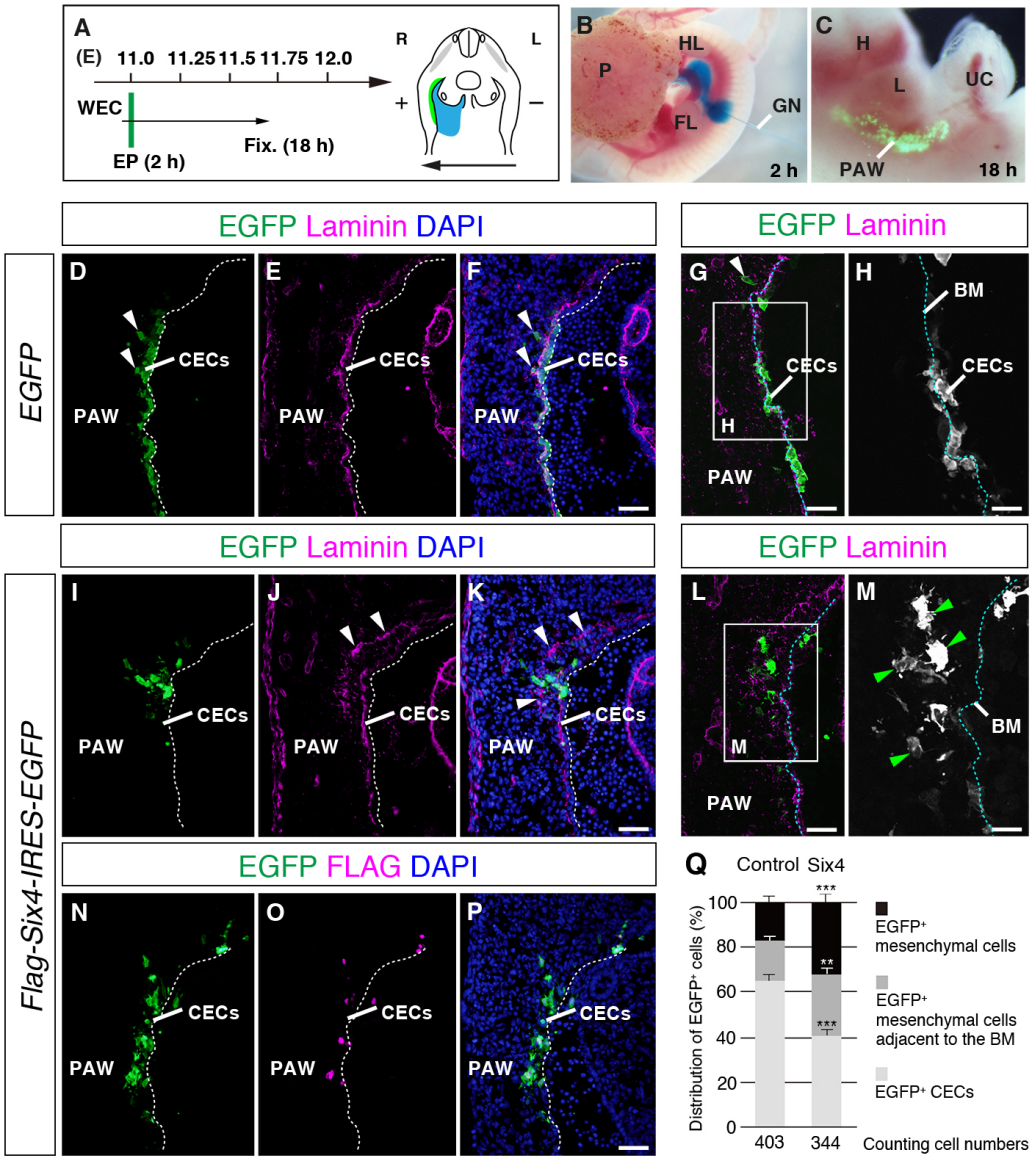
**Distribution of EGFP-labeled cells after *Six4* overexpression in abdominal CECs.** (A-C) Timescale (A) and method (B) for gene transfer into the PAW in cultured mouse embryos. DNA solution (blue) is injected into the coelomic cavity using a fine glass needle, followed by electroporation (B). EGFP expression in the PAW of the electroporated embryos is visualized at 16 h after electroporation (C). (D-H) Distribution of EGFP-positive cells in the PAW of wild-type embryos electroporated with EGFP expression plasmids. White dotted lines in D-F indicate the apical border of the CE. Most EGFP-positive cells are observed in the CE (D) and reside adjacent to the laminin-positive basement membrane (E,F). Confocal microscopy of the PAW in wild-type embryos electroporated with EGFP expression plasmids (G,H). The EGFP signal in the rectangle in G is shown in gray scale. Blue dotted lines in G, H indicate the boundary between the mesenchyme and the CE. Cells expressing EGFP in the mesenchyme region reside close to the boundary (G,H). A small number of EGFP-positive cells localizes in the mesenchymal region (arrowheads in D,F,G). (I-P) Distribution of EGFP-positive cells in the PAW of wild-type embryos electroporated with *pCAGIG-flag-Six4* plasmids. Expression of exogenous SIX4 protein is detected using a Flag antibody (O). Laminin distribution is irregular in the region distant from the normal position of the boundary between the mesenchymal region and the CE (arrowheads in J,K). Confocal microscopy of the PAW of wild-type embryos electroporated with *Six4* expression plasmids (L,M). The EGFP signal in the rectangle in L is shown in gray scale (M). Most of the cells with exogenous SIX4 protein are positioned in the mesenchymal region and show cell morphology with filopodia (green arrowheads in M). White dotted lines in I-K,N-P indicate the edge of the apical side of the CE. Blue dotted lines in L and M indicate the border between the mesenchyme and the CE. (Q) Quantification of distribution of EGFP-positive (EGFP^+^) cells electroporated with control and *Six4*-expression plasmids. A total of 403 and 344 cells are counted in control- and *Six4*-overexpressing samples, respectively. The percentages of distribution of EGFP^+^ cell types were indicated. *Six4*-overexpression in CECs decreases the ratio of EGFP^+^ cells in the CE and increases that of EGFP^+^ mesenchymal cells, compared to those in the control experiment. **P<0.01, ***P<0.001, Student's *t*-test, *n*=3. BM, basement membrane; FL, forelimb; GN, glass needle; H, heart; HL, hindlimb; L, liver; P, placenta, PAW, primary abdominal wall; UC, umbilical cord. Scale bars: 50 μm in D-G,I-L,N-P; 25 μm in H,M.

As loss of *Six4* and *Six5* caused the inefficient transition of CECs into rounded mesothelial progenitor cells in the PAW (Fig. 6), we questioned whether *Six4* induces any cellular behavioral changes in CECs. To address this issue, we overexpressed exogenous *Six4* in CECs of the PAW in cultured mouse embryos at E11.0. We injected a DNA solution containing *EGFP* expression plasmids into the coelomic cavities of cultured wild-type mouse embryos and introduced exogenous genes into CECs by electroporation (Fig. 7A,B). EGFP-positive cells were detected in the dorsal region of the PAW (Fig. 7C) 16 h after electroporation (Fig. 7A). Most of the EGFP-positive cells were localized in the inner coelomic surface of the embryos that were electroporated only with *EGFP* plasmids, and small numbers of EGFP-positive cells were observed in the mesenchymal region of the PAW (*n*=2, Fig. 7D-H). In contrast, after introduction of Flag-tagged *Six4*, EGFP-positive SIX4-overexpressing cells were detected in the mesenchymal region underneath the laminin-positive basement membrane (*n*=2, Fig. 7I-P) and exhibited a mesenchymal cell morphology with filopodia (Fig. 7M). Furthermore, ectopic distribution of laminin was noted in the mesenchyme surrounding the SIX4-overexpressing cells (Fig. 7J-L). For quantification of the effect of *Six4* overexpression in CECs, we analyzed the distribution of EGFP-positive cells in the PAW after introduction of a control vector (*pCAGIG*) or *Six4*-expression vector (*pCAGIG-flag-Six4*). In the control vector introduction, 64.9±3.1%, 17.9±2.1% and 17.2±2.4% of EGFP-positive cells were detected in the CE, in the mesenchymal region adjacent to the laminin-positive basement membranes and in the mesenchymal region, respectively (*n*=3, Fig. 7Q). Following the introduction of the *Six4*-expression vector, 40.7±3.3%, 27.3±2.6% and 31.9±2.9% of EGFP-positive cells were detected in the CE, in the mesenchymal region adjacent to the laminin-positive basement membranes and in the mesenchymal region (*n*=3, Fig. 7Q). Exogenous SIX4 decreased the ratio in the CE (*P*=6.38E-06, Student's *t*-test, *n*=3) but increased the ratio in the mesenchymal region adjacent to the laminin-positive basement membranes (*P*=0.0092, Student's *t*-test, *n*=3) and mesenchymal region (*P*=0.00053, Student's *t*-test, *n*=3) (Fig. 7Q). These results suggest that SIX4 is sufficient to promote ingression of CECs into the mesenchymal region in the PAW.

## DISCUSSION

### *Six4/Six5* double-deficient mice as a suitable animal model for human middle-type omphalocele

Various gene mutations cause body wall closure defects in mice (Brewer and Williams, 2004b) and, to date, at least 20 genes have been identified to be associated with large or small middle-type omphalocele formation (see also Table S3). However, in most of these mutant mice, anomalies other than omphalocele, such as neural tube defects, cleft palate and limb malformation, were observed but defects in the PAW have not been examined (summarized in Table S3). In the present study, we found that *Six4^−/−^;Six5^−/−^* mice show severe ventral body wall closure defects with few other anomalies, they have defects in PAW formation, and that omphalocele formation is in complete penetrance (Fig. 1, Table S2). Therefore, we emphasize that *Six4^−/−^;Six5^−/−^* mice are a suitable animal model for large and small middle-type omphalocele reproducing typical human omphalocele.

**Fig. 8.**
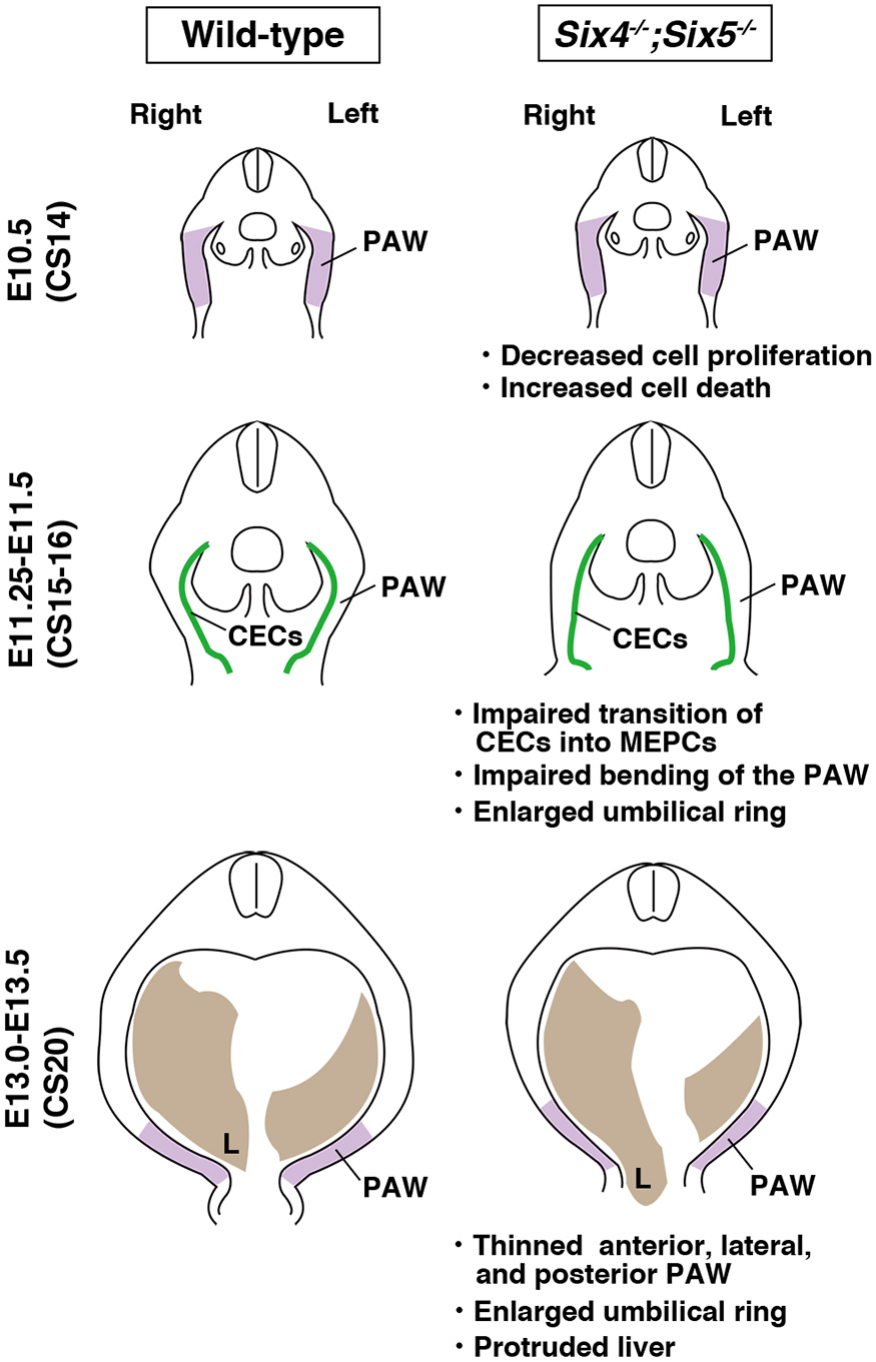
**Summary of ventral body** **wall closure in wild-type and *Six4^−/−^;Six5^−/−^* embryos.** Schematic of the normal ventral body wall closure (left) and large middle-type omphalocele formation (right) during the E10.5-E13.5 period. See text for details. The number under each mouse stage indicates the Carnegie stage (CS) of the human embryo corresponding to the mouse development stage. L, liver; MEPCs, mesothelial progenitor cells.

Notably, the phenotypes of previously identified mouse mutants with ventral body wall closure defects have not been fully compared with omphalocele in newborn humans. Our analyses revealed that the AnPAW, LaPAW and PoPAW were thinner in *Six4^−/−^;Six5^−/−^* embryos than in *Six5^−/−^* embryos at the 0% position at E13.0 when the liver was partially protruded (illustrated in Fig. 8). In contrast, the thickness at the 50% position of AnPAW and LaPAW, but not PoPAW, was larger (Fig. 2). These observations indicate that the defects in the abdominal wall region of *Six4^−/−^;Six5^−/−^* embryos ranged from anterior to posterior levels. Because co-expression of *Six4* and *Six5* was not observed in the PAW at E11.5-E12.5 before myoblast migration into the PAW (Fig. 4, Fig. S7), the thinner abdominal wall in *Six4^−/−^;Six5^−/−^* embryos was thought to be attributable to defects in the PAW before E11.5. Indeed, we detected the early occurrence of structural anomalies in *Six4^−/−^;Six5^−/−^* embryos through high-resolution micro-CT analysis. The umbilical ring of *Six4^−/−^;Six5^−/−^* embryos was larger than that of wild-type embryos before myoblast migration, suggesting that the initial change in ventral body wall closure had already occurred at E11.5 (Fig. 3). The PAW of wild-type embryos was bent at the distal levels, but that of *Six4^+/−^;Six5^−/−^* embryos was straight. As morphological differences in the PAW of other mutant mice that exhibit middle-type omphalocele have not been reported (Table S3), analyses of *Six4^−/−^;Six5^−/−^* mice first revealed that the impaired morphogenesis of the PAW may lead to middle-type omphalocele.

### Cell proliferation control during PAW formation by SIX4 and SIX5

We analyzed cell kinetics in the developing PAW of wild-type and *Six4^−/−^;Six5^−/−^* embryos at E10.5 before the onset of morphological change of the PAW. We observed the differential cell proliferation rates between the left and right somatic mesoderm-derived cells in the PAW of wild-type embryos at E10.5 (Fig. 5). Interestingly, in *Six4^−/−^;Six5^−/−^* embryos, the cell proliferation rate was significantly lower than that of wild-type embryos only in the right side of the PAW, and the rate was similar between the right and left sides, in contrast to the higher rate observed in the right than left side of wild-type embryos. In contrast, the proliferation rate of the surface ectodermal cells was lower both in the right and left PAW of *Six4^−/−^;Six5^−/−^* embryos. This indicates that *Six4* and *Six5* promote proliferation of somatic mesoderm-derived cells in the right side and proliferation of surface ectodermal cells in both sides in wild-type embryos. As our previous study revealed that *Six4* and *Six5* promote differentiation and inhibit proliferation in cultured muscle satellite cells (Yajima et al., 2010), *Six4* and *Six5* function in a context-dependent manner at embryonic and adult stages.

The growth of tissues is regulated by the balance between cell proliferation and apoptosis. Apoptotic cells were also detected in somatic mesoderm-derived cells and surface ectodermal cells of the PAW in a left-right asymmetrical manner (Fig. 5O). However, the percentage of apoptotic cells was around 3-7% in surface ectodermal cells and 2-4% in somatic mesoderm-derived cells (Fig. 5P), whereas that of EdU-positive proliferating cells was around 29-34% in surface ectodermal cells and 52-56% in somatic mesoderm-derived cells in wild-type embryos (Fig. 5P). These observations suggest that the growth rate of the PAW is mostly dependent on cell proliferation and the effects of apoptosis is marginal. Therefore, the decreased cell proliferation rate would be the main cause of the impaired growth of the PAW in *Six4^−/−^;Six5^−/−^* embryos (Fig. 8).

We observed asymmetrical growth patterns in the PAW of mouse embryos at E10.5 (Fig. 5), consistent with the previous reports of asymmetrical cell proliferation rate in the right and left PAW of early mouse embryos (Miller and Runner, 1978; Miller and White, 1998). It is possible that disruption of asymmetrical cell proliferation may affect normal asymmetrical morphogenesis of the PAW during early development. In this context, it should be noted that mice deficient of *Pitx2*, which is expressed in left somatic mesodermal cells at the early stage of E8.0-E9.0 (Campione et al., 1999), show severe ventral body wall closure defects including omphalocele (Kitamura et al., 1999; Gage et al., 1999; Eng et al., 2012). However, the cell proliferation rate in the PAW has not been examined in the *Pitx2* mutants. It is also notable that humans with Rieger syndrome carrying a mutation in *PITX2* show a higher prevalence of omphalocele than the general population (Katz et al., 2004). Therefore, disruption of the asymmetrical growth pattern in the right and left PAW may cause the impaired umbilical formation and ventral body wall closure defects.

### Implications of impaired mesothelium formation for omphalocele in *Six4^−/−^;Six5^−/−^* mice

In humans, it is proposed that the umbilical ring is formed by the body wall expanding from four different directions into the central region (Duhamel, 1963). Defects in the formation of lateral folds are thought to cause middle-type omphalocele, with loss of the anterior abdominal wall. However, how migration and growth of mesodermal cells proceed to form each PAW is unknown even in rodent species, because of the difficulty of cell lineage tracing of PAW cells at the different positions using direct-labeling methods *in utero*. Interestingly, a lineage tracing study using a *Cre-loxP* system revealed that abdominal CECs contribute to body wall cells in mice (Ariza et al., 2016). However, cell differentiation properties in the PAW at the early stages, the timing of EMT and the fates of abdominal wall CECs after ingression remain uncharacterized in detail. Therefore, we analyzed the cell differentiation properties of CECs during PAW formation in wild-type mice.

The distribution of PODXL in abdominal CECs shifts from the apically restricted pattern at E10.5 to a uniform peripheral distribution pattern at E11.25 in wild-type embryos (Fig. 6). In contrast, the apical distribution of PODXL in CECs remained apical in *Six4^−/−^;Six5^−/−^* embryos at E11.25 (Fig. 6). Furthermore, the distribution pattern of laminin produced by CECs was less fragmented in the PAW of *Six4^−/−^;Six5^−/−^* embryos compared with that of wild-type embryos (Fig. 6). These results suggest the defective transition of CECs into mesothelial progenitor cells in *Six4^−/−^;Six5^−/−^* embryos, and strongly suggest that *Six4* and *Six5* also regulate differentiation of abdominal CECs, in addition to the cell kinetics of PAW cells (Fig. 8).

Interestingly, knockout mice of *Podxl* and calreticulin (*Calr*), genes which are involved in the regulation of EMT in cultured cell lines (Meng et al., 2011; Hayashida et al., 2006), show middle-type omphalocele (Doyonnas et al., 2001; Mesaeli et al., 1999; Rauch et al., 2000). This implies that the mesothelium formation is a crucial step for proper body wall closure. Therefore, it is important to examine whether disruption of the regulation of mesothelium formation and EMT is a feature that is common to mutant mice with small and large omphaloceles.

Considering that the CECs differentiate into vascular smooth muscle cells, fibroblasts and endothelial cells in the developing lung and gut through EMT (Que et al., 2008; Wilm et al., 2005), the polarized abdominal CECs would also transit into mesothelial progenitor cells through EMT. Overexpression of *Six4* in the CECs of cultured mouse embryos promoted ingression of these cells into the mesenchymal region (Fig. 7M) and the cells became mesenchymal in shape, with filopodia, suggesting the induction of EMT. In this context, it should be noted that SIX1 and SIX4 contribute to the regulation of metastasis and EMT in cancer cells (Micalizzi et al., 2009; Li et al., 2017; Ono et al., 2012). In terms of a possible mechanism underlying the defects in the anterior abdominal wall and the enlarged umbilical ring in *Six4^−/−^;Six5^−/−^* embryos, it is plausible that the defective transition of CECs into mesothelial progenitor cells and impaired ingression of CE-derived cells into the mesenchymal region are also involved in the impaired growth of the PAW in *Six4^−/−^;Six5^−/−^* embryos (Fig. 8). However, further studies are needed to compare the cell dynamism of CECs in wild-type and *Six4^−/−^;Six5^−/−^* embryos during E10.5-E11.25 to understand the cellular mechanisms of abdominal body wall formation.

## MATERIALS AND METHODS

### Animals

Animal experiments were carried out in a humane manner after receiving approval from the Institutional Animal Experiment Committee of Jichi Medical University and in accordance with the Institutional Regulations for Animal Experiments and Fundamental Guidelines for Proper Conduct of Animal Experiment and Related Activities in Academic Research Institutions under the jurisdiction of the Ministry of Education, Culture, Sports, Science and Technology in Japan (MEXT). *Six4*- and *Six5*-deficient mice have been previously described (Ozaki et al., 2001; Klesert et al., 2000). *Six4^+/−^* and *Six5^+/−^* mice were backcrossed with C57BL/6J over 10 generations (CLEA Japan, Inc.). PCR-based genotyping was performed as previously described (Yajima et al., 2010). Midday on the day the vaginal plug was identified was designated E0.5. ICR mouse embryos (CLEA Japan, Inc.) were used for electroporation experiments.

### Histology and skeletal staining

For Hematoxylin staining, E18.5 fetuses were embedded in paraffin and cut into 8-μm sections using a microtome (SM 2000R, Leica). For Hematoxylin or Hematoxylin & Eosin staining for fetuses, frozen sections were prepared using cryostats (CM 3500S and CM1850, Leica). For skeletal staining, fetuses were fixed in 4% paraformaldehyde (PFA)/phosphate buffered saline (PBS) at 4°C for a week, and then the skin, mesenchyme and internal organs were removed. Fetuses were dehydrated in ethanol and incubated in acetone for 1 day. After incubation in ethanol, fetuses were incubated in Alcian Blue solution [0.01% Alcian Blue 8GX (Sigma), 80% ethanol, 20% acetic acid] at 37°C overnight and hydrated. Fetuses were incubated in 0.5% trypsin diluted with sodium borate solution for 6 h at 37°C and subsequently incubated in Alizarin Red S (Sigma) diluted with 0.5% KOH at room temperature overnight. Stained fetuses were washed with 0.5% KOH and treated with glycerol.

### Immunostaining

The immunostaining of frozen sections was performed as described previously (Kawasaki et al., 2016). Mouse embryos and fetuses were fixed in 4% PFA/PBS overnight at 4°C. Frozen sections were prepared using cryostats (CM 3500S and CM1850, Leica). E18.5 fetuses were embedded in paraffin and cut into 8 μm sections using a microtome (SM 2000R, Leica). Sections were blocked with 2% goat serum/TBST (Tris-buffered saline containing 0.1% Tween 20). The following primary antibodies were used: MYOD1 rabbit (1:500, C-20 sc-304, Santa Cruz), MYOG mouse (1:200, clone F5D, ab1835, Abcam), MyHC-II mouse (1:1000, clone MY-32, M4276, Sigma-Aldrich), laminin rabbit (1:5000, L9393, Sigma-Aldrich), PODXL goat (1:2000, AF1556, R&D Systems), SIX4 guinea pig (1:1000, Yajima et al., 2010), SIX5 rabbit (1:500, Yajima et al., 2010), SIX1 guinea pig (1:5000, Kawasaki et al., 2016), FLAG mouse (1:1000, clone M2, Sigma-Aldrich) and GFP chicken (1:2000, ab13970, Abcam). To detect MYOG and MyHC-II in frozen sections, antigen retrieval was performed in 0.01 M citric acid (pH 6.0) using a microwave, and a MOM kit (Vector) was used to decrease the background for endogenous IgG. For enzymatic detection, goat anti-rabbit IgG-biotin and anti-mouse IgG-biotin conjugate (E0432 and E0433, Dako) antibodies were used as secondary antibodies (1:600). For fluorescent detection, goat anti-guinea pig IgG-Alexa Fluor 488 (A11073, Invitrogen), goat anti-guinea pig IgG-Alexa Fluor 546 (A11074, Invitrogen), donkey anti-rabbit IgG-Alexa Fluor 555 (A31572, Invitrogen), donkey anti-goat IgG-Alexa Fluor 488 (705-545-147, Jackson ImmunoResearch), donkey anti-goat IgG-Alexa Fluor 555 (ab150134, Abcam) and donkey anti-chick IgY-Alexa488 (703-545-155, Jackson ImmunoResearch) conjugated antibodies were used at a 1:2000 dilution. For enzymatic color reactions, a Vectastain Elite ABC kit (Vector Laboratories) and 3,3′-diaminobenzidine tetrahydrochloride hydrate (DAB, Dojin) were used, and sections were mounted with glycergel mounting medium (Dako). For immunofluorescence, sections were counterstained with DAPI (Sigma-Aldrich) and mounted with Vectashield (Vector Laboratories). For quantification in Fig. 2D,L,N,O,Q, the sections obtained from three fetuses (*Six5^−/−^*: AnPAW level, 13 sections; LaPAW level, 14-17 sections; PoPAW level, nine sections. *Six4^−/−^;Six5^−/^*^−^: AnPAW level, 12 sections; LaPAW level, 20 sections; PoPAW level, six sections) were stained with anti-laminin antibody and DAPI. The distance of the PAW between the midline or distal position of the PAW and the RA was measured using cellSens software (Olympus), then the midline (or distal), half and proximal positions were defined as the 0%, 50% and 100% positions, respectively.

### Micro-CT imaging

Imaging procedures were performed as previously reported (Tamura et al., 2013). Embryos with the yolk sac and placenta were carefully dissected from the uterus. A small piece of the yolk sac was used for genotyping. The embryos were fixed with Bouin's fixative at 4°C overnight and then stored in 70% ethanol until treatment with reagents was performed. The embryos of each genotype were scanned using a ScanXmate-E090S scanner (Comscan Techno). Three-dimensional images and sections were analyzed using OsiriX (www.osirix-viewer.com) and 3D Slicer (www.slicer.org) software.

### *In situ* hybridization

*In situ* hybridization was performed on 12 μm frozen sections as described previously (Nonomura et al., 2010). DIG-labeled RNA probes for *Six4* and *Six5* (Nonomura et al., 2010) and *Pitx2* (a gift from H. Shiratori and H. Hamada, RIKEN Center for Biosystems Dynamics Research) were used for hybridization.

### Cell proliferation and cell death assays

A cell proliferation assay using EdU was performed as previously reported (Kawasaki et al., 2016). To label proliferating cells, EdU solution diluted in PBS (5 mg/ml) (Invitrogen) was injected into pregnant mice intraperitoneally at 50 mg/kg. Embryos were collected 30 min later and fixed in 4% PFA/PBS at 4°C overnight. Incorporated EdU was detected in 10 μm frozen sections by using Click-iT EdU-Alexa Fluor 488 (Invitrogen). To detect apoptotic cells, TUNEL was performed as previously reported (Takahashi and Osumi, 2002). Eighteen sections (EdU) and 33-35 sections (TUNEL) from three wild-type and three *Six4^−/−^;Six5^−/−^* embryos were used to obtain counts of EdU-positive cells and TUNEL-positive cells in the left and right PAW regions.

### Gene transfer into cultured mouse embryos

Whole-embryo culture and electroporation were performed according to a protocol described previously for rat embryos (Takahashi et al., 2008). Embryo dissection was modified to easily address the right side of the PAW. *pCAGIG-flag-Six4* was generated by inserting Flag-tagged mouse *Six4* cDNA into multi-cloning sites of *pCAGIG,* which contain the *CAG* promoter and IRES-EGFP (a gift from T. Matsuda and C. Cepko, Harvard Medical School; Matsuda and Cepko, 2004). After pre-culture for 2 h, *pCAX-EGFP* (Takahashi et al., 2008) or *pCAGIG-flag-Six4* (2.5 μg/μl in PBS) was injected into the coelomic cavity of the right side in E11.0 mouse embryos using a fine glass needle (G-1, Narishige International). For quantification, EGFP-positive cells were counted in the total of 20 or 21 sections obtained from three embryos in control and *Six4* experiments, respectively. The embryos were electroporated in a 20 mm×20 mm electro-chamber using an electroporator (CUY21, BEX) (70V, 950 msec intervals, 5× square pulses) and further cultured for 16 h for analyses.

### Image collection

Images were taken using microscopes (BX51 and SZX16, Olympus) equipped with a CCD-camera (DP71, Olympus), a confocal laser microscope (FV1000, Olympus), a virtual slide microscope (VS120-L100, Olympus) and a microscope (SZX16, Olympus) equipped with an HD-color camera (CSD240, Ikeda) and a recorder (VISK IR-100, Chunichi Denshi).

## Supplementary Material

Supplementary information
