# Supplementary material for: Mice doubly deficient in Six4 and Six5 show ventral body wall defects reproducing human omphalocele
Source: Dis Model Mech. 2018 Oct 25;11(10):dmm034611. doi: 10.1242/dmm.034611 (PMC6215434; doi:10.1242/dmm.034611)
Supplement: Supplementary information [file dmm-11-034611-s1.pdf]

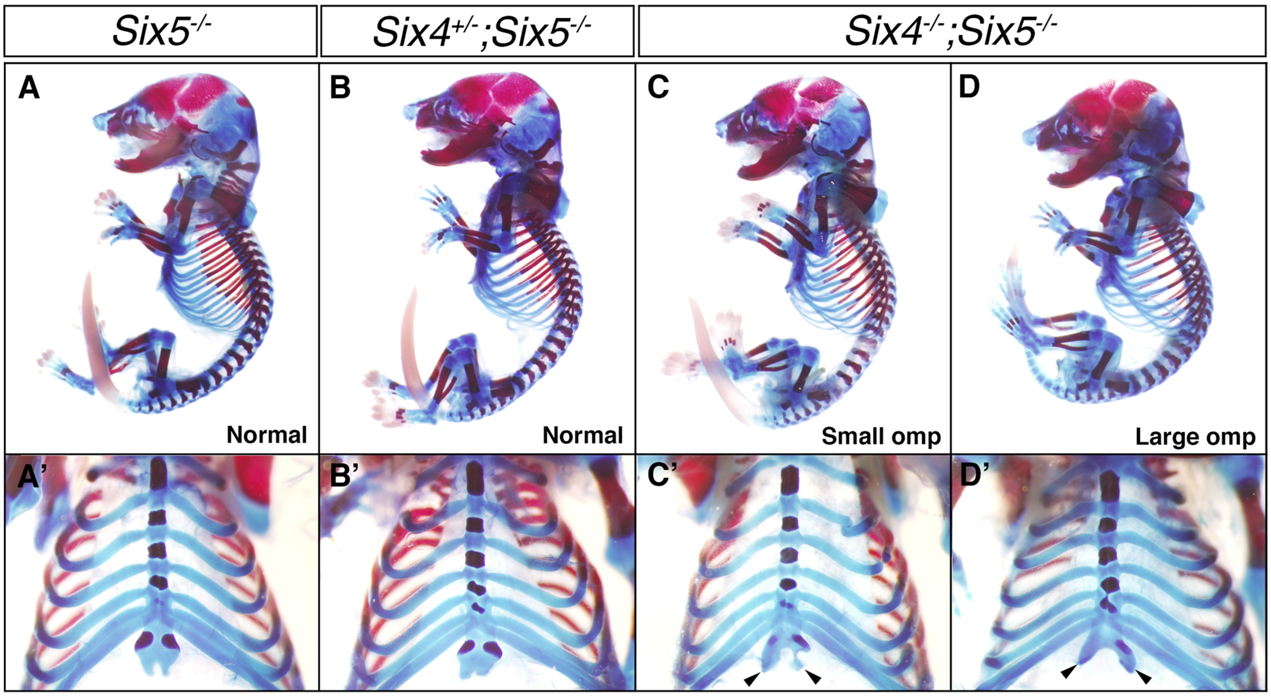

Fig. S1. Thoracic skeletal structure in *Six5$^{-/-}$*, *Six4$^{+/-}$;Six5$^{-/-}$* and *Six4$^{-/-}$;Six5$^{-/-}$* fetuses. (A-D') Thoracic skeletal structure of E18.5 *Six5$^{-/-}$* (A, A'), *Six4$^{+/-}$;Six5$^{-/-}$* (B, B') and *Six4$^{-/-}$;Six5$^{-/-}$* fetuses with small omphalocele (omp) (C, C'), and *Six4$^{-/-}$;Six5$^{-/-}$* fetuses with large omphalocele (omp) (D, D'). Bones and cartilage are stained with alizarin red and alcian blue, respectively. To clearly visualize sternums, a piece of Whatman paper was inserted under the sternums. *Six4$^{-/-}$;Six5$^{-/-}$* fetuses with small or large omphalocele show normal numbers of ribs and sternums (C', small omp, n=2/2; D', large omp, n=4/4), but the xiphoid processes bifurcate abnormally (arrowheads in C', D') compared to those of *Six5$^{-/-}$* fetuses (A').

Disease Models & Mechanisms • Supplementary information

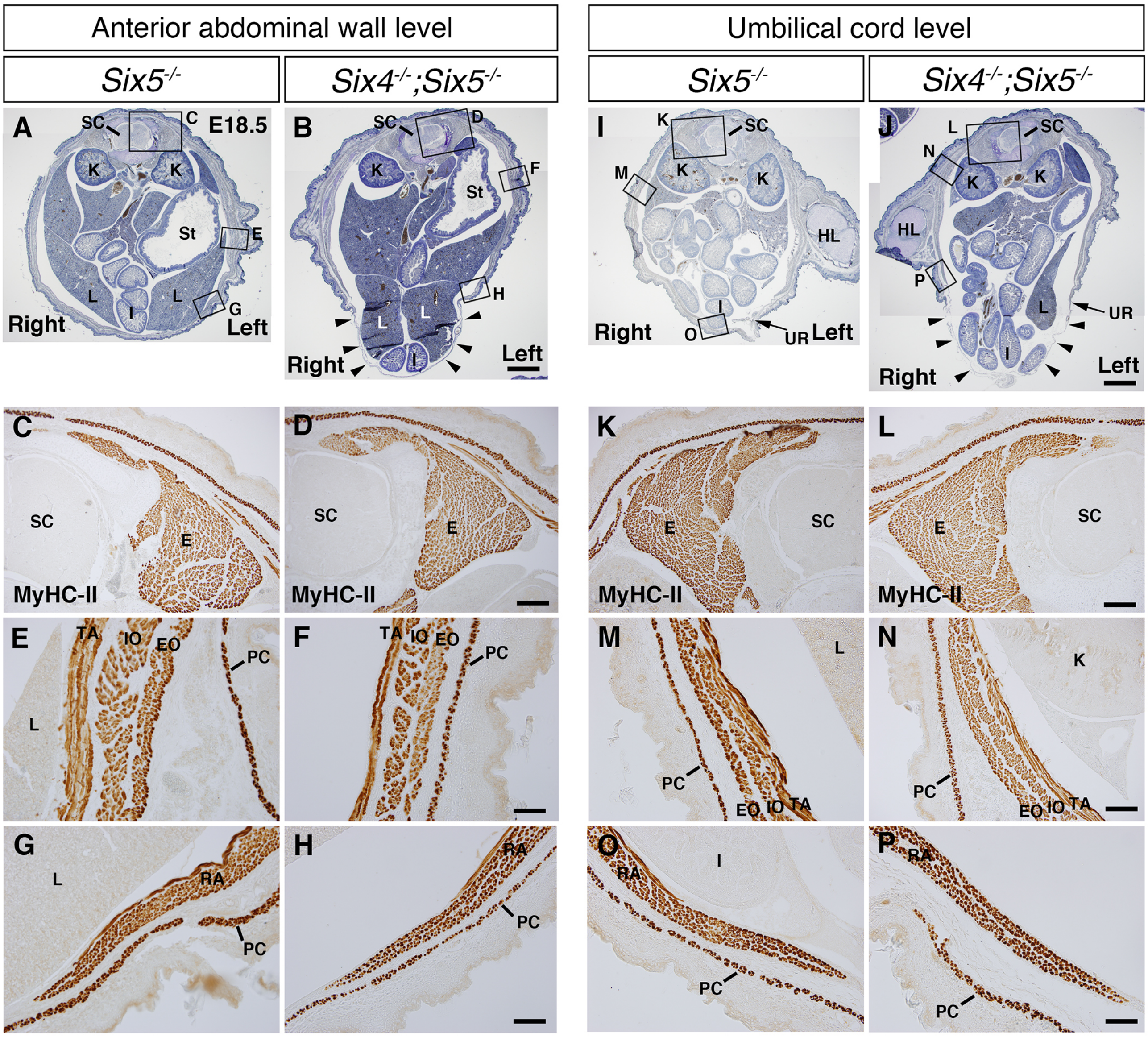

Fig. S2. Organization of abdominal muscles in *Six5*$^{-/-}$ and *Six4*$^{-/-}$*;Six5*$^{-/-}$ fetuses at E18.5. (A-P) Hematoxylin staining of paraffin sections taken from the anterior abdominal wall level (A, B) and the umbilical ring level (I, J) of E18.5 *Six5*$^{-/-}$ and *Six4*$^{-/-}$*;Six5*$^{-/-}$ fetuses with large omphalocele, respectively. Protruded liver and intestine are covered with the peritoneum and amnion (arrowheads in B, J). Localization of MyHC-II in abdominal wall muscles (C-H, K-P). Each panel corresponds to the area surrounded with rectangles in A, B, I, and J. Orientations of the fibers of four abdominal skeletal muscles, specifically the transversus abdominis muscle (TA), internal oblique muscle (IO), external oblique muscle (EO), and rectus abdominis muscle (RA), in *Six4*$^{-/-}$*;Six5*$^{-/-}$ fetuses were similar to those in *Six5*$^{-/-}$ fetuses (E-H, M-P). Extensor muscle (E) and panniculus carnosus muscle (PC) develop similarly in *Six4*$^{-/-}$*;Six5*$^{-/-}$ fetuses compared to the *Six5*$^{-/-}$ fetuses at the anterior abdominal wall and umbilical cord levels (C-H, K-P). HL, hindlimb; I, intestine; K, kidney; L, liver; SC, spinal cord; St, stomach; UR, umbilical ring. Scale bars: 1 mm in A, B, I, J; 200 µm in C, D, K, L; 100 µm in E-H, M-P.

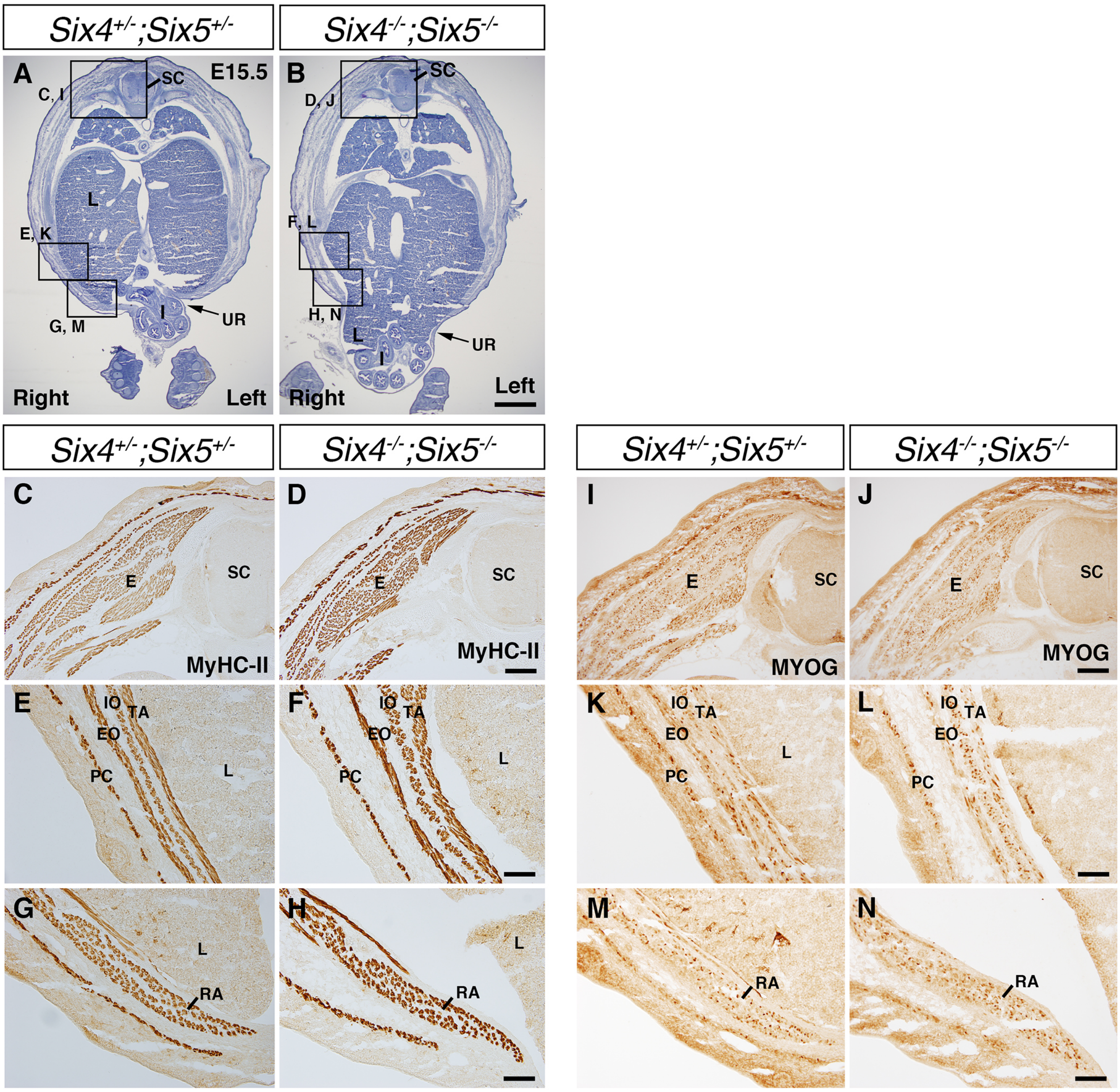

Fig. S3. Organization of abdominal muscles in *Six4*$^{+/-}$*;Six5*$^{+/-}$ and *Six4*$^{-/-}$*;Six5*$^{-/-}$ fetuses at E15.5. (A, B) Hematoxylin staining of cryosections at the umbilical ring level of *Six4*$^{+/-}$*;Six5*$^{+/-}$ and *Six4*$^{-/-}$*;Six5*$^{-/-}$ embryos. A *Six4*$^{+/-}$*Six5*$^{+/-}$ embryo shows physiological hernia (A). The liver is protruded into the umbilical cord in *Six4*$^{-/-}$*;Six5*$^{-/-}$ embryos (B). (C-H, I-N) Each panel is magnified view of the areas surrounded by rectangles in A, B. Distribution of MyHC-II (C-H) and MYOG (I-N). E, extensor muscle; EO, external oblique muscle; I, intestine; IO, internal oblique muscle; L, liver; PC, panniculus carnosus muscle; RA, rectus abdominis muscle; SC, spinal cord; TA, transversus abdominis muscle; UR, umbilical ring. Scale bars: 500 μm in A, B; 200 μm in C, D, I, J; 100 μm in E-H, K-N.

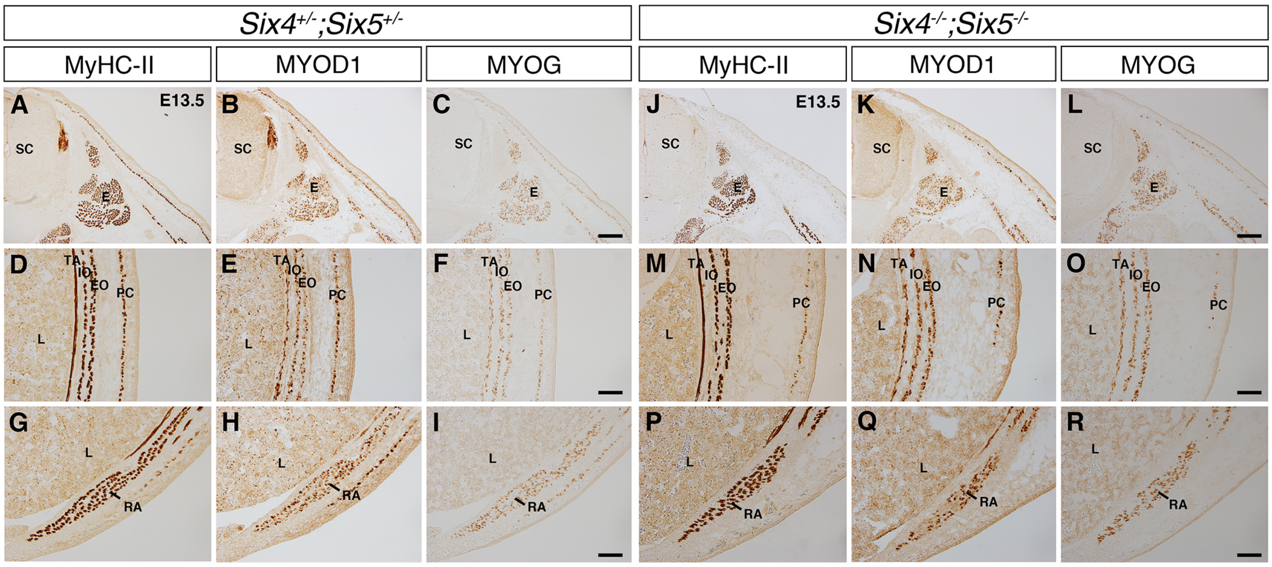

Fig. S4. Expression of transcription factors related to muscle differentiation in myoblasts of *Six4*$^{+/-}$;*Six5*$^{+/-}$ and *Six4*$^{-/-}$;*Six5*$^{-/-}$ embryos at E13.5. (A-R) Immunostaining of serial sections at the umbilical cord level in *Six4*$^{+/-}$;*Six5*$^{+/-}$ (A-I) and *Six4*$^{-/-}$;*Six5*$^{-/-}$ embryos (J-R). MyHC-II is used as a marker of skeletal muscles. MyHC-II localization patterns in extensor muscles (E) and four abdominal skeletal muscles, specifically the transversus abdominis muscle (TA), internal oblique muscle (IO), external oblique muscle (EO), panniculus carnosus muscle (PC), and rectus abdominis muscle (RA), in *Six4*$^{+/-}$;*Six5*$^{+/-}$ embryos (A, D, G) are similar to those in *Six4*$^{-/-}$;*Six5*$^{-/-}$ embryos (J, M, P). MYOD1 and MYOG are detected in abdominal skeletal muscle cells in both *Six4*$^{+/-}$;*Six5*$^{+/-}$ (B-I) and *Six4*$^{-/-}$;*Six5*$^{-/-}$ (K-R) embryos and show similar distributions except the downregulation in the PC. L, liver; SC, spinal cord. Scale bars: 200 μm in A-C, J-L; 100 μm in D-I, M-R.

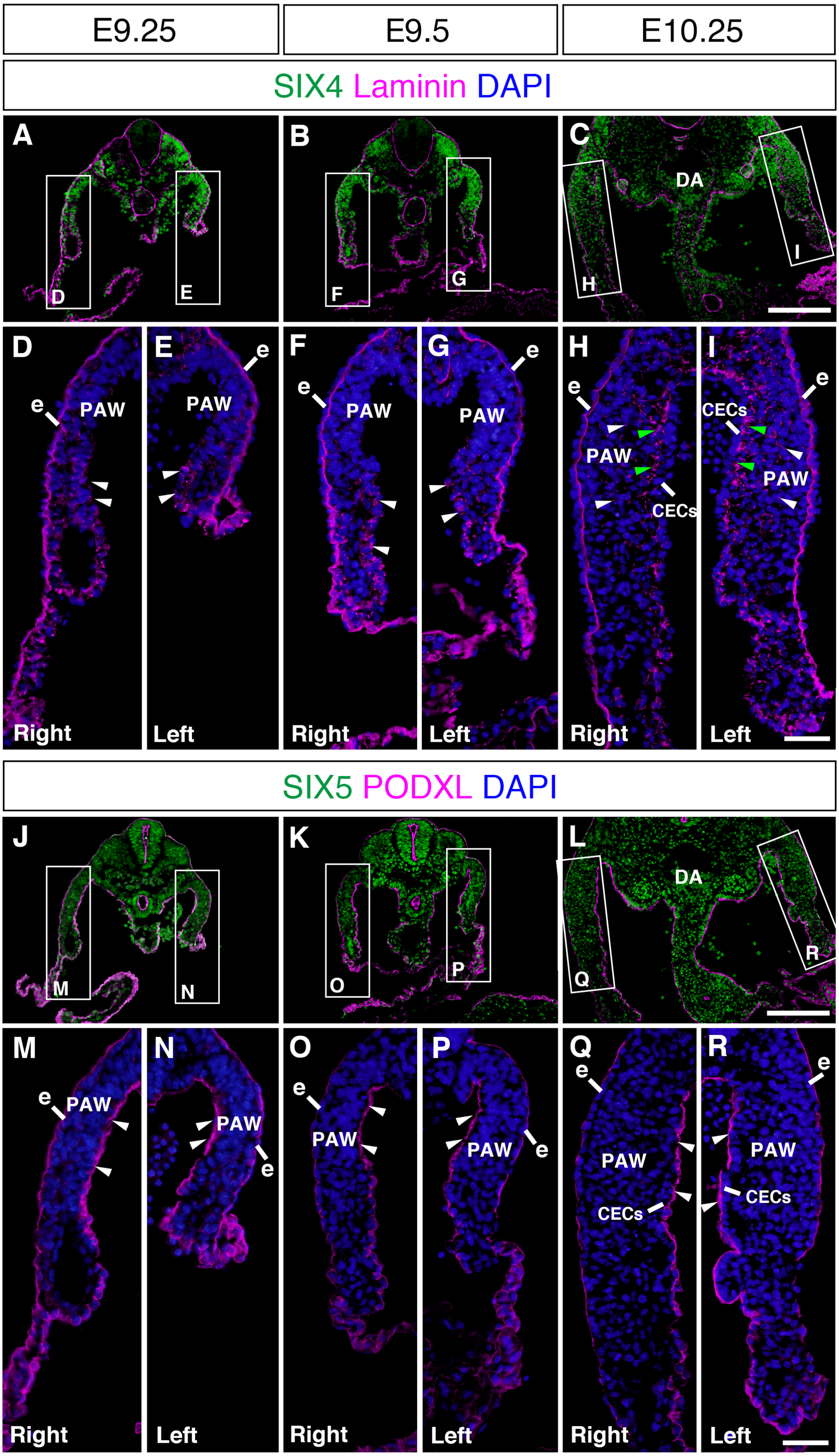
E9.25
E9.5
E10.25
SIX4 Laminin DAPI
A
B
C
DA
D
E
F
G
H
I
PAW
e
CECs
Right
Left
SIX5 PODXL DAPI
J
K
L
M
N
O
P
Q
R

Fig. S5. Localization of SIX4, SIX5, PODXL and laminin in the early PAW of wild-type embryos. (A-I) Immunostaining with SIX4 and laminin antibodies in cross sections at the level of the PAW at E9.25 (A, D, E), E9.5 (B, F, G), and E10.25 (C, H, I). SIX4 is detected in the PAW though E9.25-E10.25 (A-C). Laminin accumulates in the basal side of surface ectodermal cells but not in the mesenchymal region of the PAW at E9.25 and E9.5. Scattered distribution of laminin is observed in the ventral region adjacent to the blood vessels at E9.25 and E9.5 (white arrowheads in D-G). Scattered distribution of laminin is observed in the mesenchymal region of the PAW (white arrowheads in H, I), and accumulation of laminin is detected in the border between the mesenchyme and the CE of the PAW at E10.25 (green arrowheads in H, I). (J-R) Immunostaining with SIX5 and PODXL antibodies in cross sections at the level of the PAW at E9.25 (J, M, N), E9.5 (K, O, P), and E10.25 (L, Q, R). SIX5 is detected in the PAW through E9.5-E10.25 (J-L). PODXL localizes in the apical side of CECs adjacent to the coelomic cavity (arrowheads in M-R) and the apical side of the surface ectoderm though E9.25-E10.25. Signals within the dorsal aorta (DA) are the auto-fluorescence of blood cells. CECs, coelomic epithelial cells; e, ectoderm; PAW, primary abdominal wall; Scale bars: 200 μm in A-C, J-L; 50 μm in D-I, M-R.

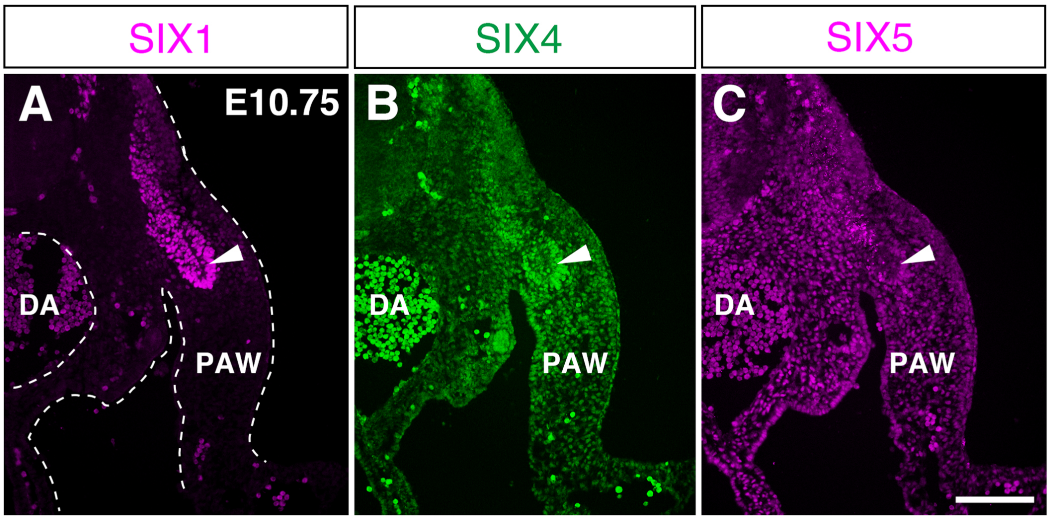

Fig. S6. Localization of the SIX1, SIX4 and SIX5 proteins in wild-type embryos. (A-C) SIX1 localizes in muscle progenitors (an arrowhead in A) but not in the PAW of wild-type embryos at E10.75. SIX4 and SIX5 localize in PAW cells and muscle progenitor cells (arrowheads in B, C). Signals within the dorsal aorta (DA) are the auto-fluorescence of blood cells. PAW, primary abdominal wall. Scale bars: 200 μm in A-C.

Disease Models & Mechanisms • Supplementary information

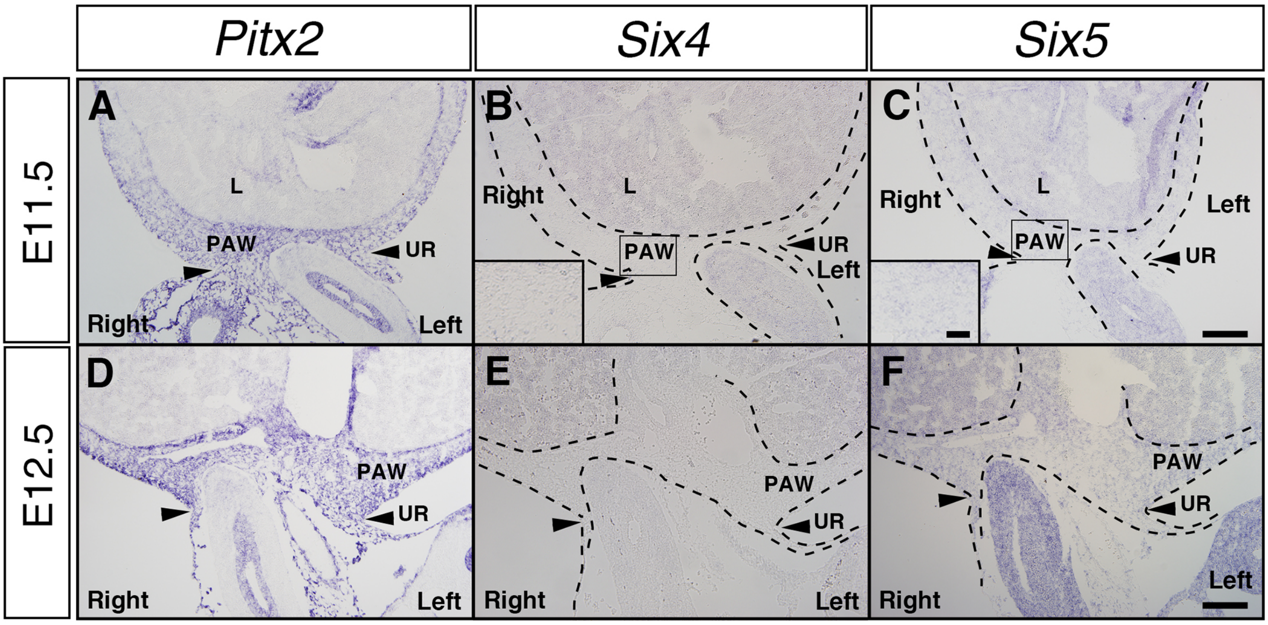

Fig. S7. Expression of *Pitx2*, *Six4* and *Six5* mRNAs in the primary abdominal wall of wild-type embryos. (A-F) *Pitx2* is used as a marker for the primary abdominal body wall (PAW). Arrowheads indicate the position of the umbilical ring (UR). At E11.5 and E12.5, *Pitx2* is expressed in the PAW (A, D), but *Six4* is not expressed in the PAW (B, E). *Six5* expression is observed in the PAW (C, F). Insets in B and C show higher magnification of the PAW in B and C, respectively. Dotted lines in B, C, E, F indicate the border of the body wall. L, Liver. Scale bars: 200 μm in A-C; 50 μm in inset in B, C; 200 μm in D-F.

Table S1. Genotypic analysis of fetuses at E18.5.

	**WT**	***Six4***$^{+/-}$	***Six4***$^{-/-}$	**Total number of fetuses**
Number of fetuses analyzed	21 (24.7%)	43 (50.6%)	21 (24.7%)	85 (from 10 litters*)
Theoretical value	25%	50%	25%	

	Six5$^{+/-}$	***Six5***$^{-/-}$	***Six4***$^{+/-}$***;Six5***$^{+/-}$	***Six4***$^{+/-}$***;Six5***$^{-/-}$	***Six4***$^{-/-}$***;Six5***$^{+/-}$	***Six4***$^{-/-}$***;Six5***$^{-/-}$	**Total number of fetuses**
Number of fetuses analyzed	14 (10.7%)	16 (12.2%)	31 (23.7%)	39 (29.8%)	16 (12.2%)	15 (11.5%)	131 (from 17 litters**)
Theoretical value	12.5%	12.5%	25%	25%	12.5%	12.5%	

	Six5$^{-/-}$	***Six4***$^{+/-}$***;Six5***$^{-/-}$	***Six4***$^{-/-}$***;Six5***$^{-/-}$	**Total number of fetuses**
Number of fetuses analyzed	11 (27.5%)	15 (37.5%)	14 (35.0%)	40 (from 6 litters***)
Theoretical value	25%	50%	25%	

Six4*$^{+/-}$ (♀) mated with *Six4*$^{+/-}$ (♂), ***Six4*$^{+/-}$*;Six5*$^{+/-}$ (♀) mated with *Six4*$^{+/-}$*;Six5*$^{-/-}$ (♂), and * *Six4*$^{+/-}$*;Six5*$^{-/-}$ (♀) mated with *Six4*$^{+/-}$*;Six5*$^{-/-}$ (♂).

Table S2. Incidence of ventral body wall closure defects in fetuses of each genotype at E18.5.

	**WT**	***Six4***$^{+/-}$	***Six5***$^{+/-}$	***Six4***$^{+/-}$***;Six5***$^{+/-}$	***Six5***$^{-/-}$	***Six4***$^{-/-}$	***Six4***$^{+/-}$***;Six5***$^{-/-}$	***Six4***$^{-/-}$***;Six5***$^{+/-}$	***Six4***$^{-/-}$***;Six5***$^{-/-}$
Numbers of fetuses analyzed	21	43	13	27	24	21	46	16	28
Normal umbilical ring	21 (100%)	43 (100%)	13 (100%)	26 (96.3%)	24 (100%)	17 (81.0%)	39 (84.8%)	2 (12.5%)	0 (0%)
Enlarged umbilical ring	0 (0%)	0 (0%)	0 (0%)	1 (3.7%)	0 (0%)	4 (19.0%)	5 (10.9%)	9 (56.3%)	0 (0%)
Small omphalocele	0 (0%)	0 (0%)	0 (0%)	0 (0%)	0 (0%)	0 (0%)	2 (4.3%)	3 (18.8%)	9 (32.1%)
Large omphalocele	0 (0%)	0 (0%)	0 (0%)	0 (0%)	0 (0%)	0 (0%)	0 (0%)	2 (12.5%)	19 (67.9%)

All fetises were obtained from 10 litters of **Six4*$^{+/-}$ (♀) mated with *Six4*$^{+/-}$ (♂), 16 litters of *Six4*$^{+/-}$*;Six5*$^{+/-}$ (♀) mated with *Six4*$^{+/-}$*;Six5*$^{-/-}$ (♂), and 5 litters of *Six4*$^{+/-}$*;Six5*$^{-/-}$ (♀) mated with *Six4*$^{+/-}$*;Six5*$^{-/-}$ (♂).

Disease Models & Mechanisms • Supplementary information

Table S3. Comparison of *Six4/Six5* double deficient mice and other mutant mice with middle-type omphalocele.

Mutated gene	Protein category	Primary abdominal wall defect (E10.5~ E12.5)	Secondary abdominal wall defect (E13.5~)	Neural tube closure defects	Cleft parate	Limb malfomation	Class of middle-type omphalocele	References
Six4/Six5 (double KO)	Transcription factor	Yes	No	No	No	No	large & small	This study
Ataxin/AtaxinL1 (double KO)	DNA binding protein	n.d.	n.d.	Yes, hydrocephalus	n.d.	No	large	Lee et al. (2011). *Dev Cell* **21**, 746-757.
Calr (calreticulin)	Cellular protein	n.d.	n.d.	No	n.d.	No	small	Mesaeli et al. (1999). *J Cell Biol* **144**, 857-868. Rauch et al. (2000). *Exp Cell Res* **256**, 105-111.
efnb1	Ligand	n.d.	n.d.	No	n.d.	Yes	large	Compagni et al. (2003). *Dev Cell* **5**, 217-230.
Ephb2/Ephb3 (double KO)	Receptor	n.d.	n.d.	No	n.d.	No	large	Dravis and Henkemeyer (2011). *Dev Biol* **355**, 138-151.
Fgfr1/Fgfr2 (double cKO using Rosa Esr-Cre)	Receptor	n.d.	Yes, disruption of demis, muscle and connective tissues	No	n.d.	No	small	Nichol et al. (2011). *J Pediatr Surg* **46**, 90-96.
Flna (Filamin A)	Celluar protein	n.d.	n.d.	No	n.d.	No	large	Lian et al. (2012). *J Neurosci* **32**, 7672-7684.
Gad67	Enzyme	n.d.	n.d.	No	Yes	No	small	Oh et al. (2010). *PLoS One*, **5**, e9758. Saito et al. (2010). *Mol Brain* **3**, 40. Kakizaki et al. (2015). *Neuroscience* **288**, 86-93
Gad67/Gad57 (double KO)	Enzyme	n.d.	n.d.	No	Yes	No	small	Kakizaki et al. (2015). *Neuroscience*, **288**, 86-93.
Hic1	Zinc finger BTB domain protein	n.d.	n.d.	Yes	Yes	No	small	Carter et al. (2000). *Hum Mol Genet* **9**, 413-419.
Marcks	Protein kinase C substrate	n.d.	n.d.	Yes, anencephaly	n.d.	No	small	Chen et al. (1996). *Proc Natl Acad Sci USA*, **93**, 6275-6279.
MeKK4	Enzyme	n.d.	n.d.	Yes	n.d.	No	large	Abell et al. (2005). *Mol Cell Biol* **25**, 8948-8959. Chi et al. (2005). *Proc Natl Acad Sci USA* **102**, 3846-3851.
Nf2	Cytoskeletal protein	n.d.	n.d.	Yes	n.d.	n.d.	large	McLaughlin et al. (2007). *Proc Natl Acad Sci USA*, **104**, 3261-3266.,
Podxl (podocalyxin)	Membrane protein	n.d.	n.d.	No	n.d.	No	small	Doyonnas et al. (2001). *J Exp Med* **194**, 13-27.
Rock1	Enzyme	n.d.	Yes, epithelial cell differentiation defect	No	n.d.	No	large & small	Shimizu et al. (2005). *J Cell Biol* **168**, 941-953. Thumkeo et al. (2005). *Genes Cells* **10**, 825-834.
Rock2	Enzyme	n.d.	n.d.	No	n.d.	No	small	Thumkeo et al. (2005). *Genes Cells* **10**, 825-834.
Slc12a5 (KCC2)	Membrane transporter	n.d.	n.d.	No	n.d.	No	large	Hübner et al. (2001). *Neuron* **30**, 515-524.
Slc32a1 (VGAT)	Membrane transporter	n.d.	n.d.	No	Yes	No	large & small	Oh et al. (2010). *PLoS One* **5**, e9758. Wojcik et al. (2006). *Neuron* **50**, 575-587. Saito et al. (2010). *Mol Brain* **3**, 40. Kakizaki et al. (2015). *Neuroscience* **288**, 86-93.
Sox11	Transcription factor	n.d.	n.d.	No	Yes	Yes	large	Sock et al. (2004). *Mol Cell Biol* **24**, 6635-6644. Bhattaram et al. (2010). *Nat Commun* **1**, 9.
Tmem67	Membrane protein	n.d.	n.d.	No	n.d.	n.d.	small	Abdelhamed et al. (2015). *Dis Model Mech* **8**, 527-541.
Trip11 (GMAP210)	Golgi protein	n.d.	n.d.	No	n.d.	Yes, chondrogenesis defect	small	Follit et al. (2008). *PLoS Genet* **4**, e1000315. Smits et al. (2010). *N Engl J Med* **362**, 206-216.

n.d., no data

Disease Models & Mechanisms • Supplementary information

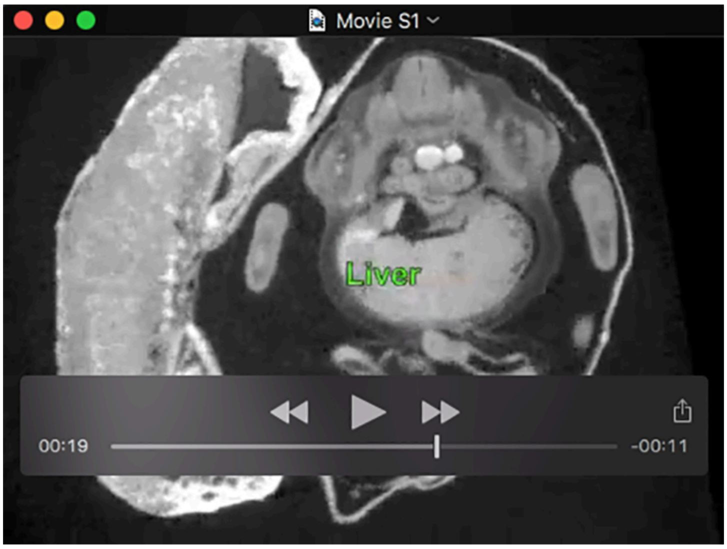

Movie S1. Micro-CT cross-sections of the E12.5 wild-type mouse embryo (related to Fig. 3B')

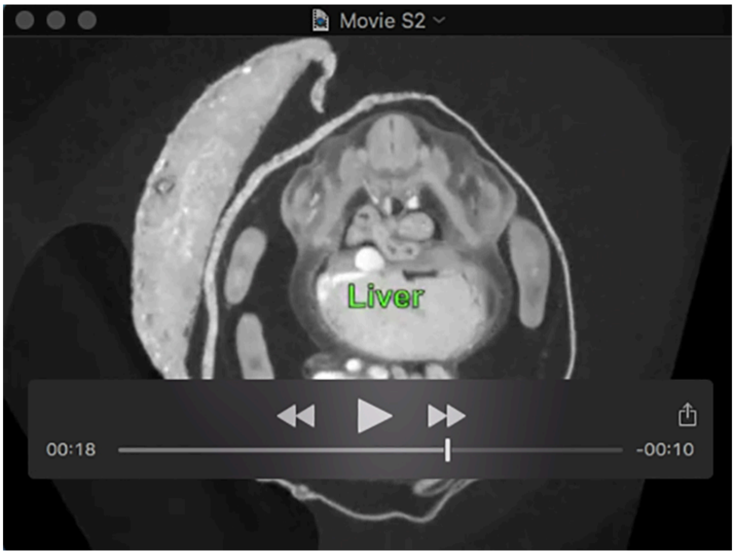

Movie S2. Micro-CT cross-sections of the E12.5 *Six4*$^{-/-}$;*Six5*$^{-/-}$ mouse embryo (related to Fig. 3D').

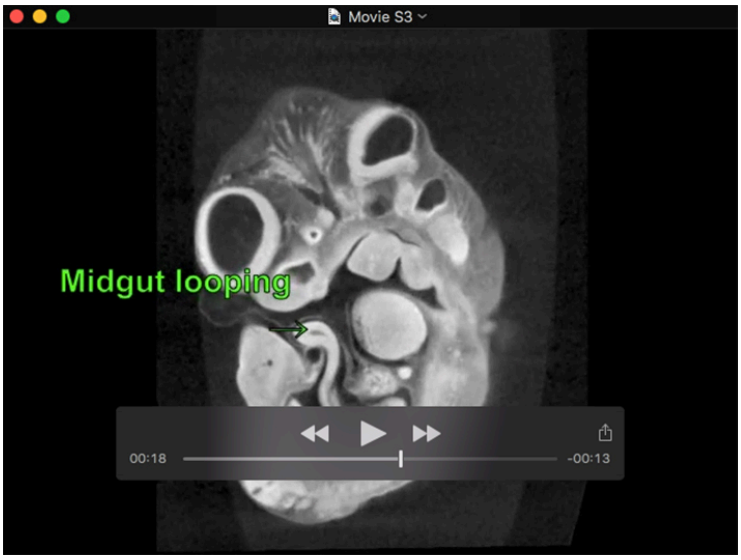

Movie S3. Micro-CT sagittal-sections of the E11.5 wild-type embryo (related to Fig. 3E).

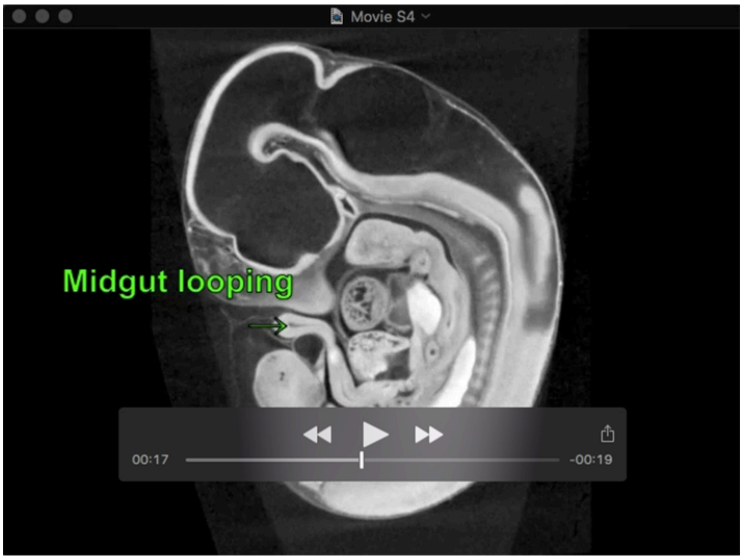

Movie S4. Micro-CT sagittal-sections of the E11.5 *Six4*$^{-/-}$;*Six5*$^{-/-}$ embryo (related to Fig. 3G).
